# Single-cell type annotation with deep learning in 265 cell types for humans

**DOI:** 10.1093/bioadv/vbae054

**Published:** 2024-04-08

**Authors:** Sherry Dong, Kaiwen Deng, Xiuzhen Huang

**Affiliations:** Skyline High School, Ann Arbor, MI 48103, United States; National AI Campus and Department of Computational Biomedicine, Cedars-Sinai Medical Center, West Hollywood, CA 90069, United States; Department of Computational Medicine and Bioinformatics, University of Michigan, Ann Arbor, MI 48109, United States; National AI Campus and Department of Computational Biomedicine, Cedars-Sinai Medical Center, West Hollywood, CA 90069, United States

## Abstract

**Motivation:**

Annotating cell types is a challenging yet essential task in analyzing single-cell RNA sequencing data. However, due to the lack of a gold standard, it is difficult to evaluate the algorithms fairly and an overfitting algorithm may be favored in benchmarks. To address this challenge, we developed a deep learning-based single-cell type prediction tool that assigns the cell type to 265 different cell types for humans, based on data from approximately five million cells.

**Results:**

We achieved a median area under the ROC curve (AUC) of 0.93 when evaluated across datasets. We found that inconsistent labeling in the existing database generated by different labs contributed to the mistakes of the model. Therefore, we used cell ontology to correct the annotations and retrained the model, which resulted in 0.971 median AUC. Our study reveals a limiting factor of the accuracy one may achieve with the current database annotation and points to the solutions towards an algorithm-based correction of the gold standard for future automated cell annotation approaches.

**Availability and implementation:**

The code is available at: https://github.com/SherrySDong/Hierarchical-Correction-Improves-Automated-Single-cell-Type-Annotation. Data used in this study are listed in Supplementary Table S1 and are retrievable at the CZI database.

## 1. Introduction

Single-cell RNA sequencing (scRNA-seq) is powerful for measuring the gene expression of many individual cells in a sample and is now widely used in all areas of biomedical research such as understanding the immune system and introducing targets for disease treatment (Tang *et al.* 2019), understanding interspecies gene transfer, and chemical communication (Website), and others. One important component in understanding scRNA-seq data is annotating cell types. However, the annotation process was manually tedious and technically challenging, requiring the biologists to spend months sorting cells, isolating cell types and profiling the molecular properties (Rust *et al.* 2006, Lubeck and Cai 2012).

Computational methods have been developed as alternative approaches to accelerate the annotation process. For example, one may use unsupervised clustering and correlation analysis (Aran *et al.* 2019, de Kanter *et al.* 2019, Hou *et al.* 2019, Proserpio 2019, Zhang *et al.* 2019a), combined with existing knowledge of marker genes (Zhang *et al.* 2019b, Cao *et al.* 2020; Shao *et al.* 2020) to annotate the cell types. Additionally, one can use supervised learning algorithms with “gold standard” annotation for the training purpose, where a variety of base learners, including random forest, boosted trees and deep neural networks had been used (Lieberman *et al.* 2018, Alquicira-Hernandez *et al.* 2019, Johnson *et al.* 2019, Tan and Cahan 2019, Xie *et al.* 2019, Website). The accuracies reported in these studies are excellent, but not perfect. For example, the scPred model17 had a mean area under the receiver-characteristic function of 96.4, the SuperCT model15 had a concordance of up to 91.4% for some cell types, and the scMatch11 model had recall rates from 79% to 99%.

Our goal is to identify the sources of the error in predicting cell types, in order to develop a method that is scalable with the growing dataset. Of note, the large size of single cell public data repository makes many base learners such as tree-based methods inappropriate. We implemented a deep learning-based classification model trained on all healthy human tissues in the Chan Zuckerberg Initiative CZ CELLxGENE Discover (CZI) database (Cellxgene Data Portal 2023). The method can handle millions of single-cell expression profiles and can predict multiple cell types (265) at the same time. We used cross-dataset validation to evaluate the model performance (to prevent substantial overestimation of prediction accuracy) and the model achieved a median area under the ROC curve (AUC) of 0.930 across datasets. We further analyzed the sources of the errors we made in the predictions and found that the hierarchical cell type structure (and synonymous cell types) was a main contributor. Correction made on this hierarchy brought the median AUC to 0.971. This work has generated an automated single-cell type classification model that accelerates the cell-type annotation of scRNA-seq data and could also help standardize the labeling of cells.

## 2. Methods

### 2.1 Data acquisition

We downloaded all normal (healthy), mature (>13 years old) human datasets with 10x 3’, 10x 3’ v1, 10x 3’ v2, 10x 3’ v3, 10x 5’ v1, 10x 5’ v2, 10x technology, and Smart-seq2 assays from the CZI database. Eighteen datasets (each containing multiple tissues/samples) met these criteria, which included 4 536 532 cells (Supplementary Table S1). As many of the datasets contained multiple assays at the same time, the model trained here is likely to be robust to many assay types. The average number of cells per dataset was 266 855, ranging from 31 691 cells to 1 248 980 cells. Tissue types include the esophagus, the spleen, the epithelium, the heart, and a wide sampling of human tissues. The files downloaded were in the h5ad format.

**Figure 1.**
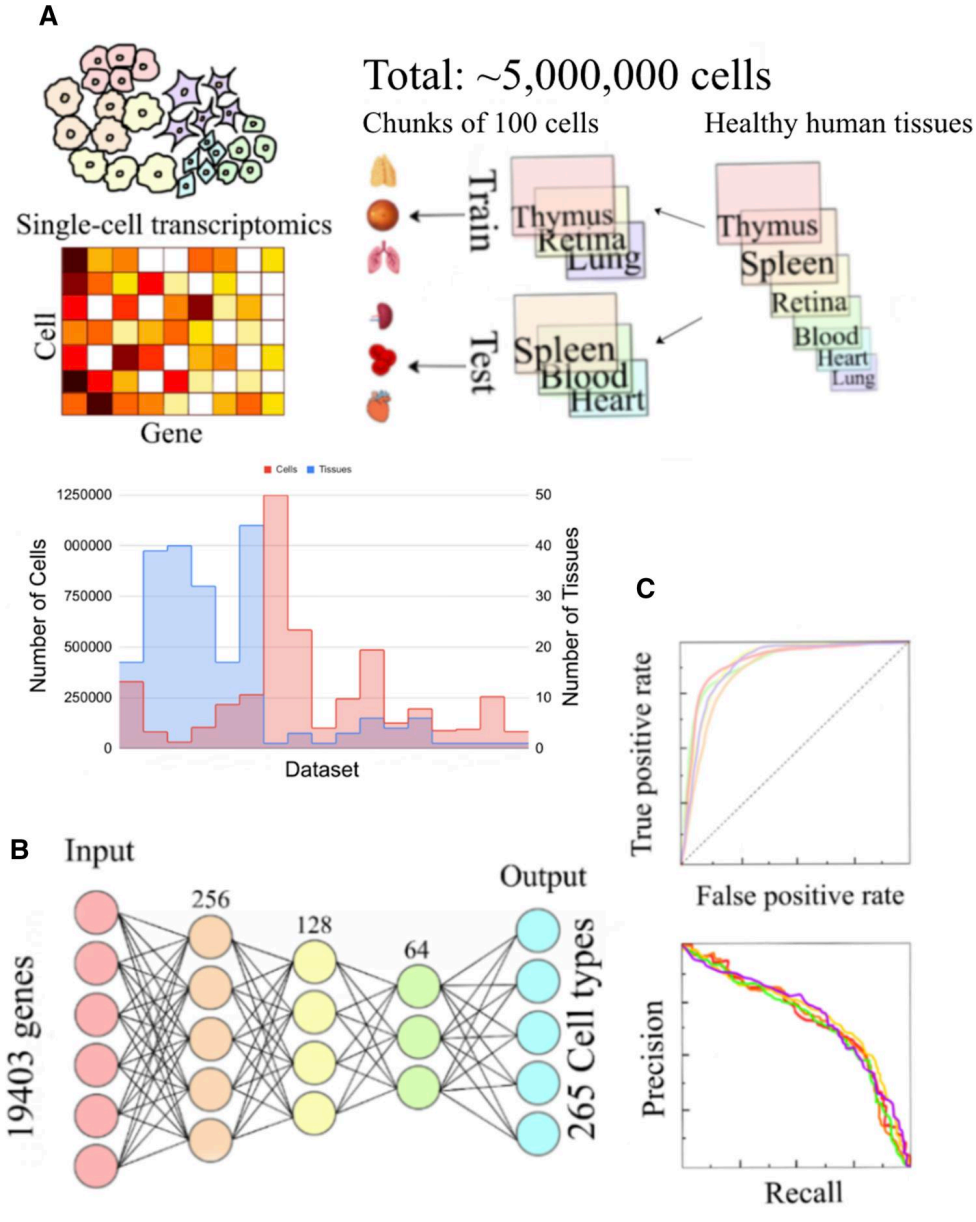
Overview of experiment design for evaluating the deep learning models. (A) Single cell data are represented as gene expression matrices, where each column is a gene and each row is a cell. The total number of cells in the data sets is about 5 million. First, the data sets are randomly split into train and test. Then the data is split into chunks of 100 cells, which allows convenient batch loading during deep learning. We show the distribution of the number of cells. (B) We used a fully-connected neural network for training cell type annotation models. Each color represents a layer. The first layer is the input, which is the 19 403 genes and the last layer is the output, which is the 265 cell types. (C) We used the AUC and AUPRC to evaluate the classification accuracy of the model.

To process the h5ad files into human-readable format, we used the anndata.read_h5ad function from the anndata Python package. We converted the files into expression matrices, where the rows are the genes and the columns are the cells. As it is impossible to load the entire data into memory, we split the files into chunks of 100 cells for the convenience of training (each batch is 100 cells) and saved the chunks into pickle files for efficient disk usage. We randomly split the data by datasets into 80% train and 20% test sets (Fig. 1B).

### 2.2 Neural network architecture

After splitting the data, we used a fully connected neural network to train the model. The input was the expression profile of 19 403 genes, which was used to predict 265 cell types. The neural network had 3 layers. The first layer had 256 neurons, the second had 128 neurons, and the third layer had 64 neurons. The learning rate was 3e-5, with Adam optimizer of beta_1 = 0.9, beta_2 = 0.999. We used cross-entropy loss (Loss Functions–ML Glossary documentation), i.e.,

-(∑┬(c=1))┴M y_(o,c) log⁡(p_(o,c))

M—number of cell types (265); log—the natural log; y—binary indicator (0 or 1) if type label c is the correct classification for the cell; p—predicted probability cell o is of type c.

The model was trained for 20 epochs and called back using a randomly split validation set.

### 2.3 Hierarchical correction of cell types

We observed that much of the poor performance in the initial model was caused by ambiguous annotations. We therefore hand-curated a list of child-parent and synonymous relationship lists of cell types based on the cell type ontology tree 19. When retraining the model, we propagated all annotations of a child to its parent cell types iteratively and propagated the synonymous annotations. This created an annotation tree for each cell, where it can be annotated to multiple cell types. This set of new annotations was used to retrain and evaluate the model.

### 2.4 Cross-validation and evaluation

AUC is the Area under the ROC Curve, which is the Receiver Operating Characteristics Curve. The ROC curve is made by plotting the false positive rate on the x-axis and the true positive rate on the y-axis (Narkhede 2018). True positive rate is the number of correct positive predictions out of all positives and false positive rate is the number of negative predictions predicted to be positive out of all negatives. The larger the AUC is, the better the performance.

$$\begin{array}{c} TPR=TP/P=TP/(TP+FN)\\ FPR=FP/N=FP/(FP+TN) \end{array}$$

AUPRC is the area under the Precision-Recall curve. The precision-recall curve shows the recall on the x-axis and the precision on the y-axis. Recall is the number of correct positive predictions out of all ground truth positives, which is the same as true positives. Precision is the number of correct positive predictions out of all positive predictions.

$$\begin{array}{c} R\mathrm{ecall}=TP/P=TP/(TP+FN)\\ P\mathrm{recision}=TP/(TP+FP) \end{array}$$

For both measurements, the evaluation is done for each cell type independently. For example, if a cell is annotated to a type A, as well as its parent A’, A and A’ are separately evaluated, if it is predicted to be associated to A’ but not A, then, for A’ this prediction is considered to be a correct case, but for A this prediction is considered to be a wrong case. The same applies to the synonym relationships.

### 2.5 Computing environment and implementation packages

We used Pickle and Pandas for data loading, and Keras and TensorFlow for deep learning networks. The code was implemented in Python 3.6. Training a single model takes approximately 5 hours and 10GB memory.

## 3. Results

### 3.1 Predicting 265 types of healthy human cells in CZI database with a neural network

We curated 18 datasets from the CZI database representing a diverse array of normal human tissues. Multiple samples exist in each dataset (248 samples in total), often coming from different tissues. In each sample, the data is a matrix containing the measured expression value of each gene for each cell. The number of cells in each data set ranges from 31 691 to 1 248 980 (median 195 395) (Fig. 1A). These datasets together contain around 5 million cells. The large dataset precludes the application of typical machine learning algorithms that need to load all feature data at once due to limitations of computer memory. As a comparison, we also implemented lightgbm MultiOutputRegressor, by selecting the number of cells in the training set that can fit into the memory, and found the performance was substantially worse (Supplementary Figs S1 and S2). Therefore, deep learning algorithms that allow loading data batch by batch is a natural option for this dataset.

**Figure 2.**
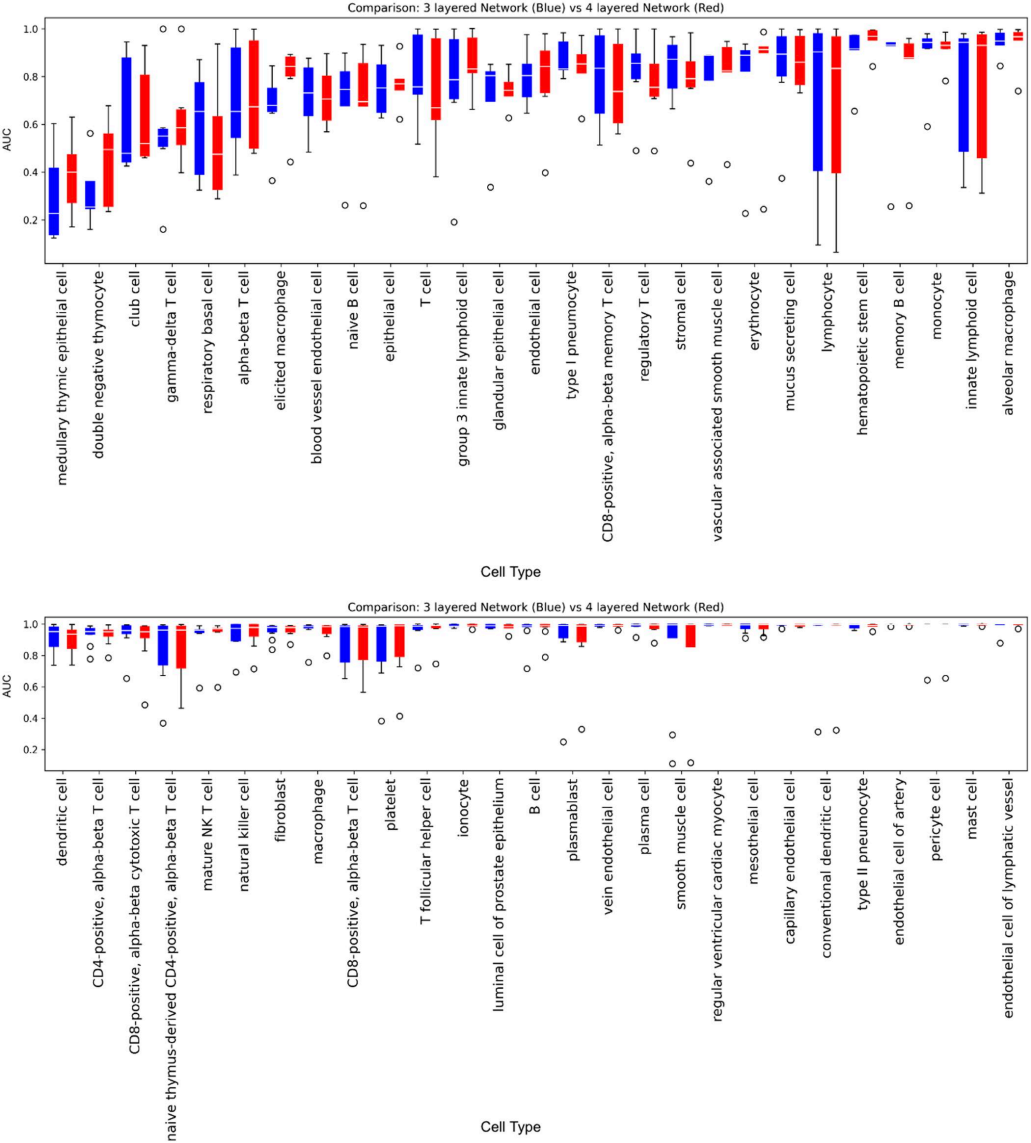
AUC comparison of 3 and 4-layer networks. We selected the cell types that are evaluated at least 5 times in the 10-fold cross-validation. The 4-layer network contains 4 hidden layers. The first layer has 512 neurons, the second has 256, the third has 128, and the forth has 64 neurons. We selected the cell types that are evaluated at least 5 times in the 10-fold cross-validation here for demonstration.

Our deep learning network is a fully connected neural network, a choice that was made to reflect the independence between genes. The network contains 3 hidden layers. The first hidden layer had 256 neurons, the second hidden layer had 128 neurons and the third hidden layer had 64 neurons (Fig. 1B). This architecture is designed to gradually reduce the dimension of the gene expression data. The input layer contains 19 403 genes and the output includes 265 distinct values allowing predicting different cell types simultaneously in a single model. For every cell, the type(s) that it is annotated to is labeled as 1 and otherwise 0. If a cell is annotated to multiple cell types after hierarchical correction (see below), there will be multiple ones in its gold standard. We compared the above model with a deeper architecture, which resulted in similar performance (Fig. 2). We trained a total of 20 epochs and retrieved the best epoch using a validation set separated from the training set (Supplementary Fig. S3).

In order to prevent overestimation of performance, during cross-evaluation, the training and testing sets are separated by datasets, instead of cells. This is because we found that cross-cell evaluation leads to a significant overestimation of the performance of an AUC > 0.99 (Supplementary Fig. S7). Therefore, if one cell of a dataset appears in the training set, then all the cells of the dataset are grouped in the training. This restricts us to evaluate only the cell types appearing in the training set, but is a more rigorous evaluation that reflects the true performance. In each cross-validation iteration, we randomly selected 20% of the datasets as test sets. As cell type assignment is a multiclass classification problem, we used AUC for evaluation (Fig. 1C).

### 3.2 Cross-validation shows strong performance but also reveals annotation inconsistently is a major contributor to mistakes

**Figure 3.**
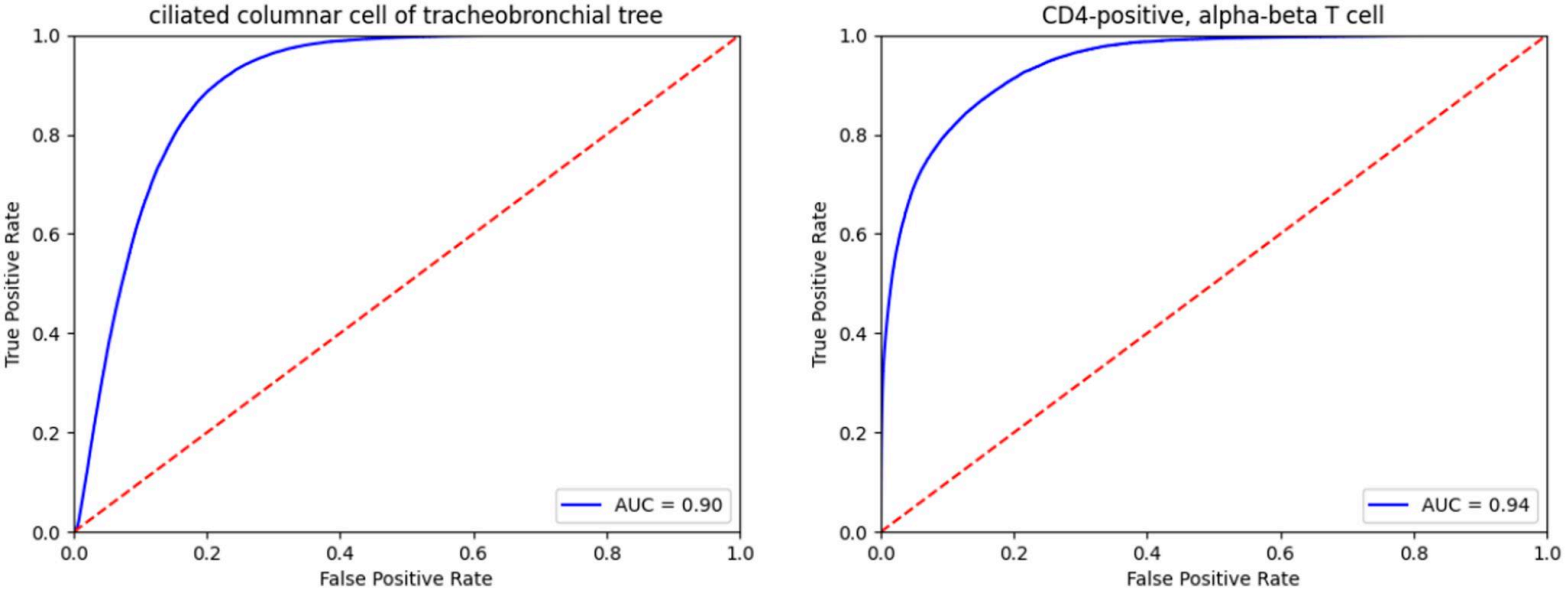
Performance represented by the area under the receiver operating characteristic (AUROC) curves for two example cell types (Ciliated columnar cell of tracheobronchial tree, and CD4-positive, alpha-beta T cell) in cross-validation.

The above deep learning model reached a median AUC of 0.930 across different cell types in cross dataset-validation (Fig. 3 for two representative cell types with an AUC of 0.90 and 0.94 respectively, Figs S4–S6). The AUC for different cell types ranged from 0.1698 to 1. Some of the best-performing cell types were plasmacytoid dendritic cell, endothelial cell of the lymphatic vessel, mast cell, natural killer cell, pericyte cell, type II pneumocyte, and conventional dendritic cell, for which the model made perfect predictions across datasets.

We next looked at the cell types that performed worst in cross-dataset predictions and identified two major issues leading to poor performance. Firstly, cells are annotated with different labeling systems across datasets. For example, in the data we used, the blood vessel endothelial cell and vein endothelial cell were labeled as different cell types, even though they are the same cell. This caused the AUC of the blood vessel endothelial cell to be estimated much lower than it actually is. We designate this type of error as the “synonym error.” Second, some broad cell types may contain multiple subtypes. For example, cells labeled as “T cell” may be subcategorized into alpha-beta T cell, and CD4-positive, etc. While some datasets annotate cells at broad levels such as “T cell,” others may annotate at lower levels. Because we are training and evaluating the types as different categories, this can result in both wrong predictions and underestimation of the performance. We designated this type of error “hierarchical error”. This type of error can be mostly captured by the cell ontology hierarchy as will be shown below. Secondly, we found that cells in transient states tend to perform worse, e.g. the precursor B cell. This is a similar issue as synonym error and hierarchical error. We consider these two types of errors can be improved by correcting and propagating the gold standard label with expert knowledge.

### 3.3 Hierarchical correction of the cell type annotation substantially improves the model performance

Recognizing the major performance imperfection could originate from ambiguous annotations, we created a table labeling the parental and synonymous relationship between terms based on the cell type ontology 19 (Supplementary Table S3). We propagated all children annotations to parent annotations and propagated all synonymous annotations mutually. Therefore, a single cell can now be annotated to multiple categories. For example, the ciliated columnar cell of the tracheobronchial tree, ciliated epithelial cell, and lung ciliated cell are children annotations to the ciliated cell and the ciliated epithelial cell would also be a child annotation to the epithelial cell. The blood vessel endothelial cells and vein endothelial cells are synonymous, so they are children annotations of each other. Compared to the old annotation system in which each cell is only annotated to a single cell type, a cell can be annotated to multiple cell types in this new system. Additionally, we used sigmoid instead of softmax as the activation function in the last layer of the neural network, the network allows a cell to be predicted to multiple cell types.

**Figure 4.**
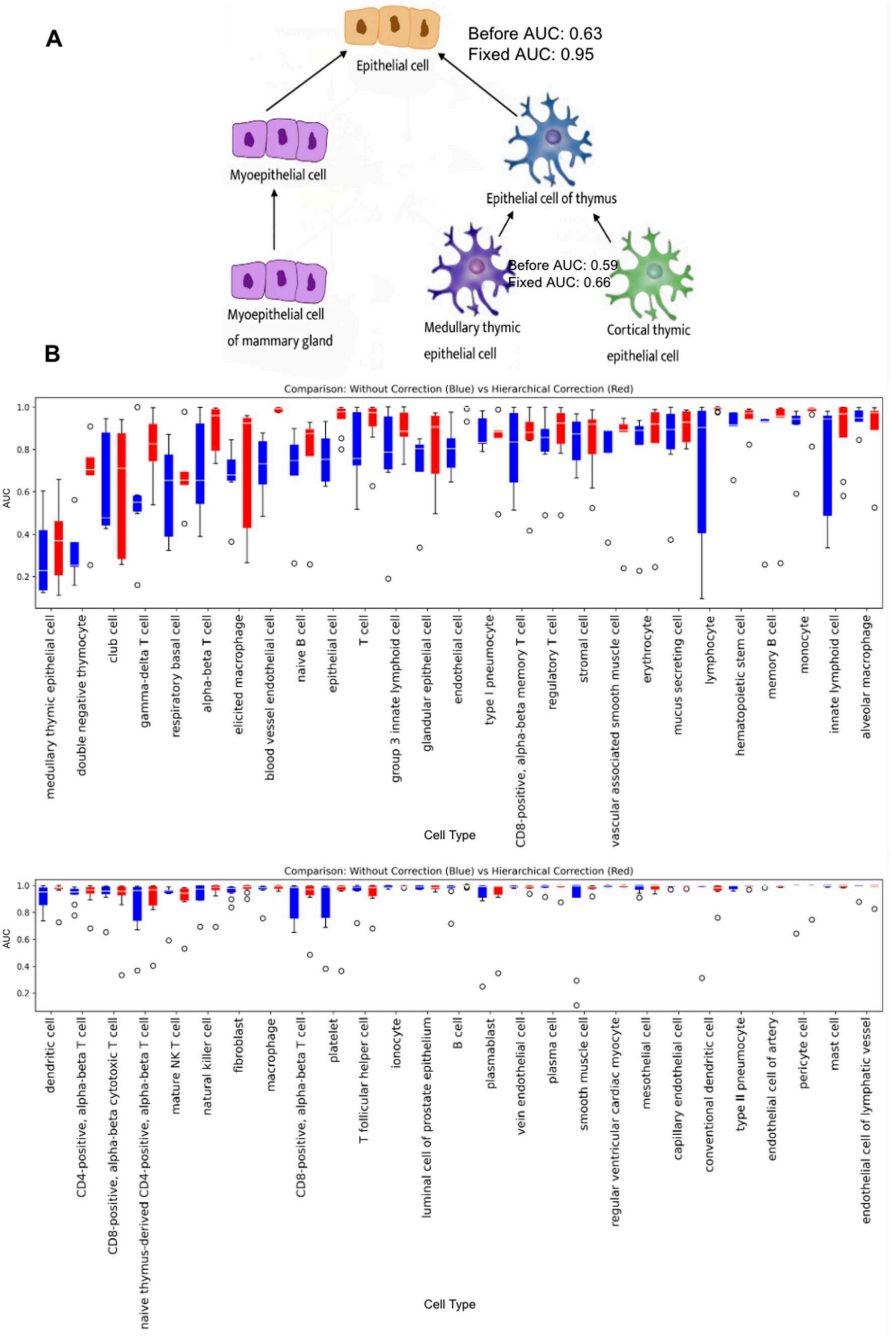
We applied hierarchical correction to the cell types, with the arrows pointing from the children's cell types to the parent cell types. (A) An example effect of this correction was depicted here. In cases of synonyms, we propagated the annotation mutually (e.g. the blood vessel endothelial cell and the vein endothelial cell are synonyms, so there are arrows pointing both ways). (B) shows the AUC of each cell type before and after hierarchical correction. We selected the cell types that are evaluated at least 5 times in the 10-fold cross-validation. The median AUC before hierarchical correction is 0.932 and after hierarchical correction is 0.971.

The median AUC improved from 0.93 to 0.971 using the propagated annotations to retrain the model. The improvement is most obvious in cell types that involve these annotation errors (Fig. 4). For example, the blood vessel endothelial cell improved from 0.705 to 0.992, and the erythrocyte improved from 0.757 to 0.920. The consistent performance improvement suggests that hierarchical correction can successfully address the issue of annotation inaccuracy in single-cell-type prediction and is an essential step in training cell-type annotation models. Furthermore, we tested on the model using the lung atlas data published in 2023, and achieved a median AUC of 0.986. For example, in classifying squamous epithelial cell, the model achieved a performance of 0.992. In classifying lung macrophage, the model achieved a performance of 0.979 (Supplementary Table S5). Additionally, we found that for cell types with a large number of cells, the performance is stably excellent. There is a weak, non-significant relationship between the number of cells of the cell type and the AUC (Supplementary Fig. S8).

## Supplementary Material

vbae054_Supplementary_Data
