# Supplementary material for: Single-cell type annotation with deep learning in 265 cell types for humans
Source: Bioinform Adv. 2024 Apr 8;4(1):vbae054. doi: 10.1093/bioadv/vbae054 (PMC11031354; doi:10.1093/bioadv/vbae054)
Supplement: vbae054_Supplementary_Data [file vbae054_supplementary_data.pdf]

Supplementary figures

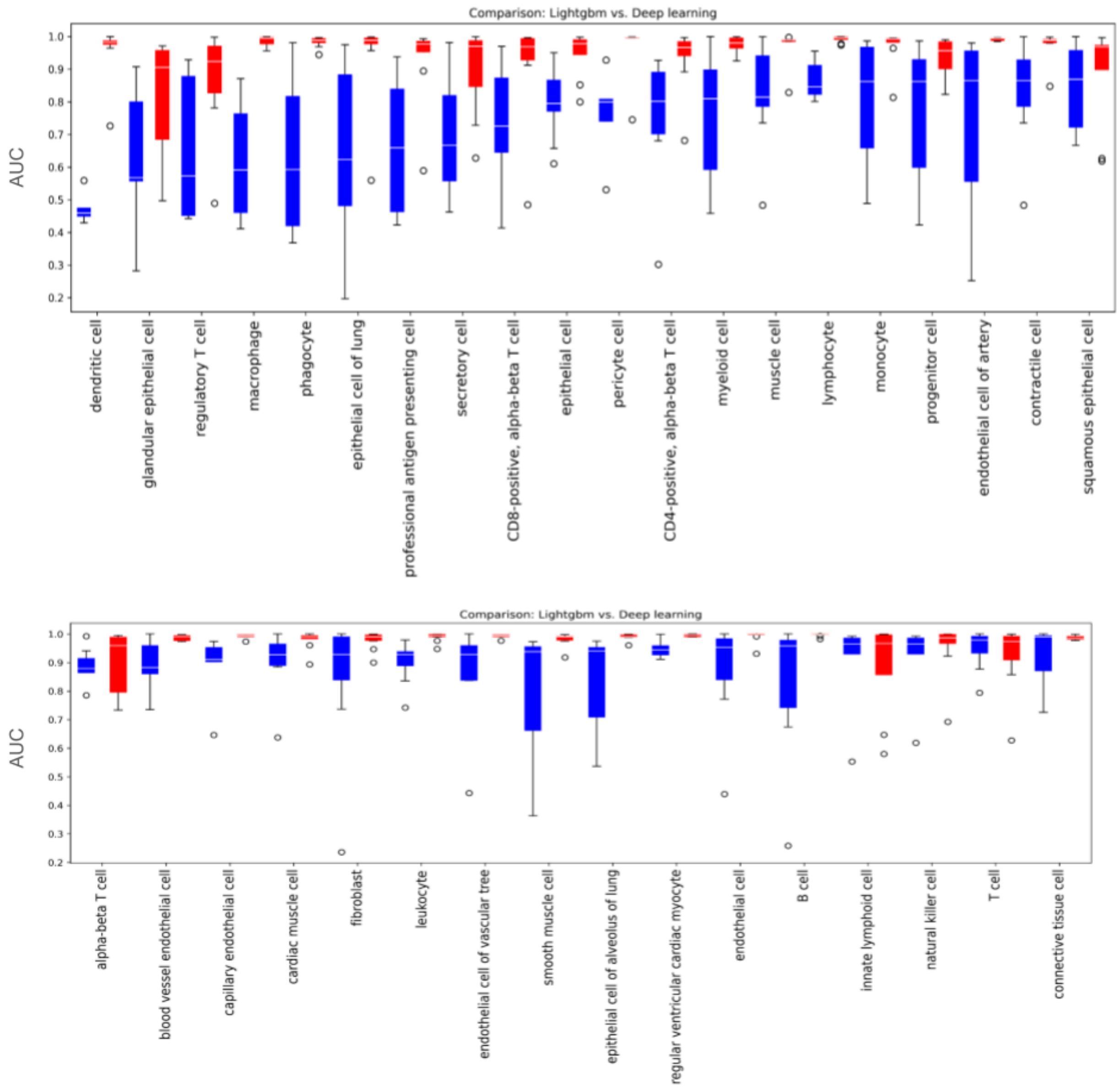

Figure S1. AUC comparison of lightGBM (MultiOutputRegressor) and deep learning model. We selected the cell types that are evaluated at least 5 times in the 10-fold cross-validation.

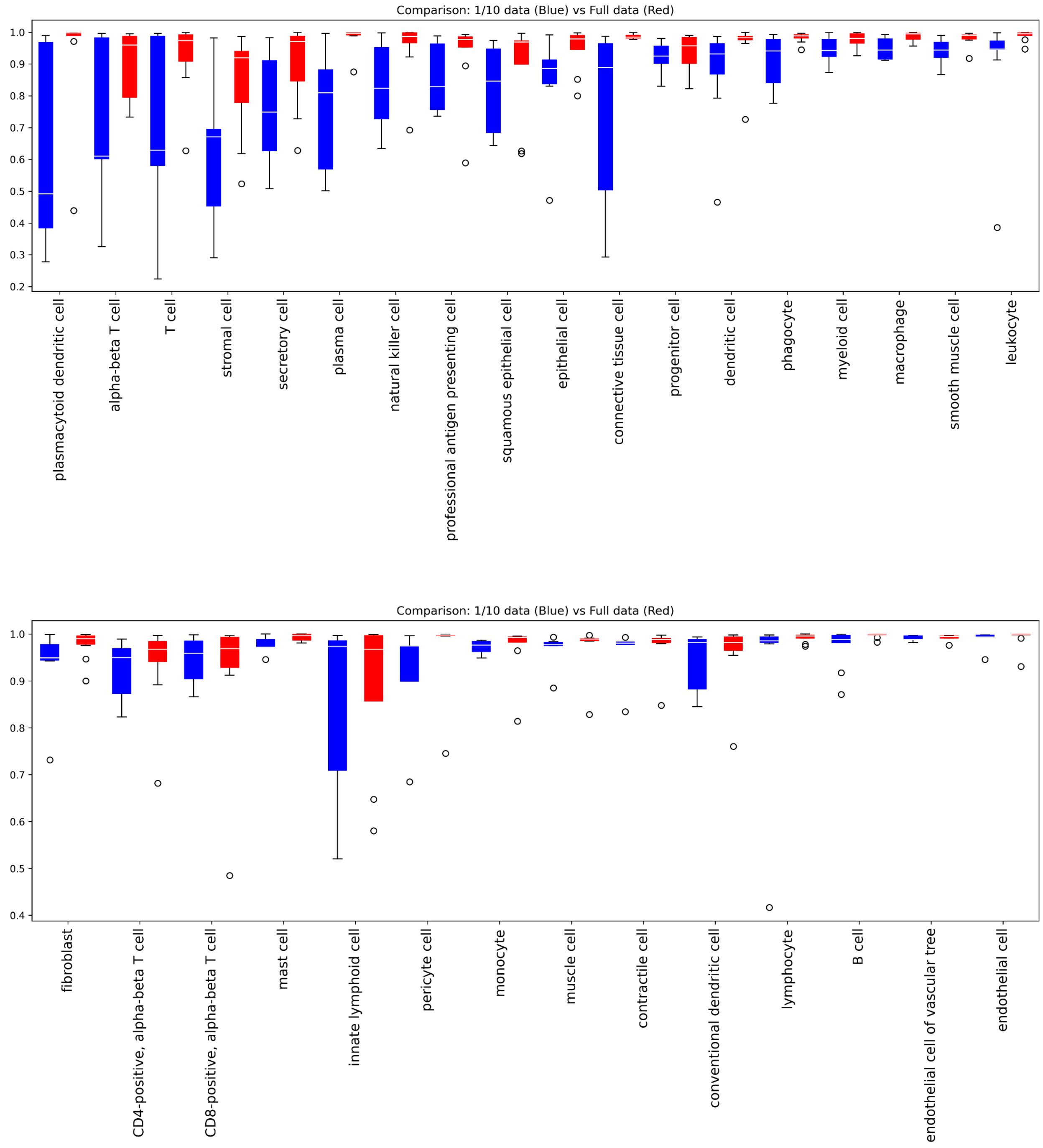

Figure S2. AUC comparison of using 1/10 of the data versus using the entire datasets using deep learning models. We selected the cell types that are evaluated at least 5 times in both 10-fold cross-validation.

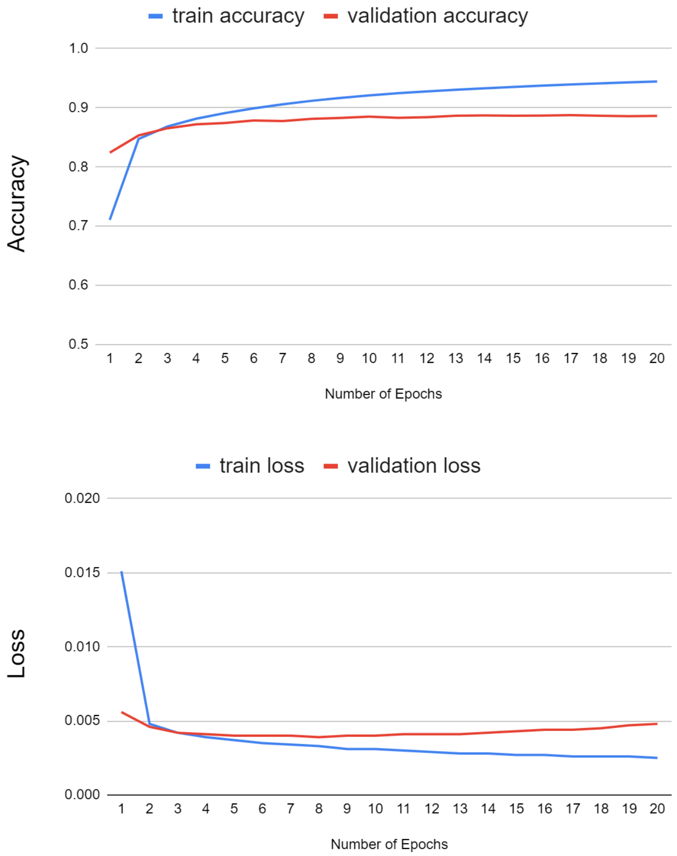

Figure S3. The graph for accuracy and loss. The train accuracy always improves, while the validation accuracy starts to improve slower at five epochs and stops improving after 10 epochs. The train loss continuously decreases. The validation loss is the lowest at about 8 epochs, indicating that the model already converged.

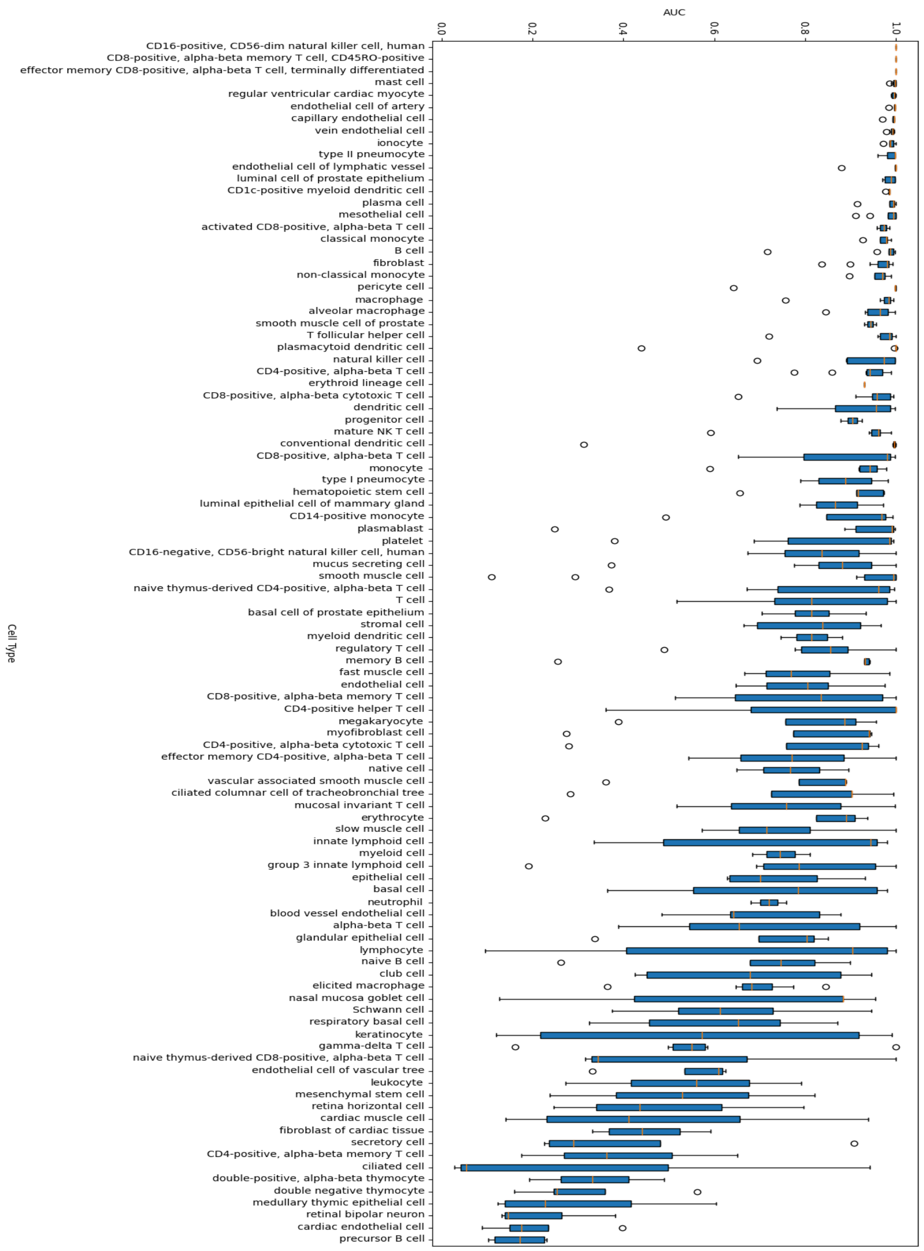

Figure S4. AUC for each cell type. The average AUC is 0.804 and median is 0.932. The cell types with the best AUC were CD16-positive, CD56-dim natural killer cell; CD8-positive, alpha-beta memory T cell; and effector memory CD8-positive, alpha-beta T cell. Some of the worst performing cell types were precursor B cell, cardiac endothelial cell, and retinal bipolar neuron.

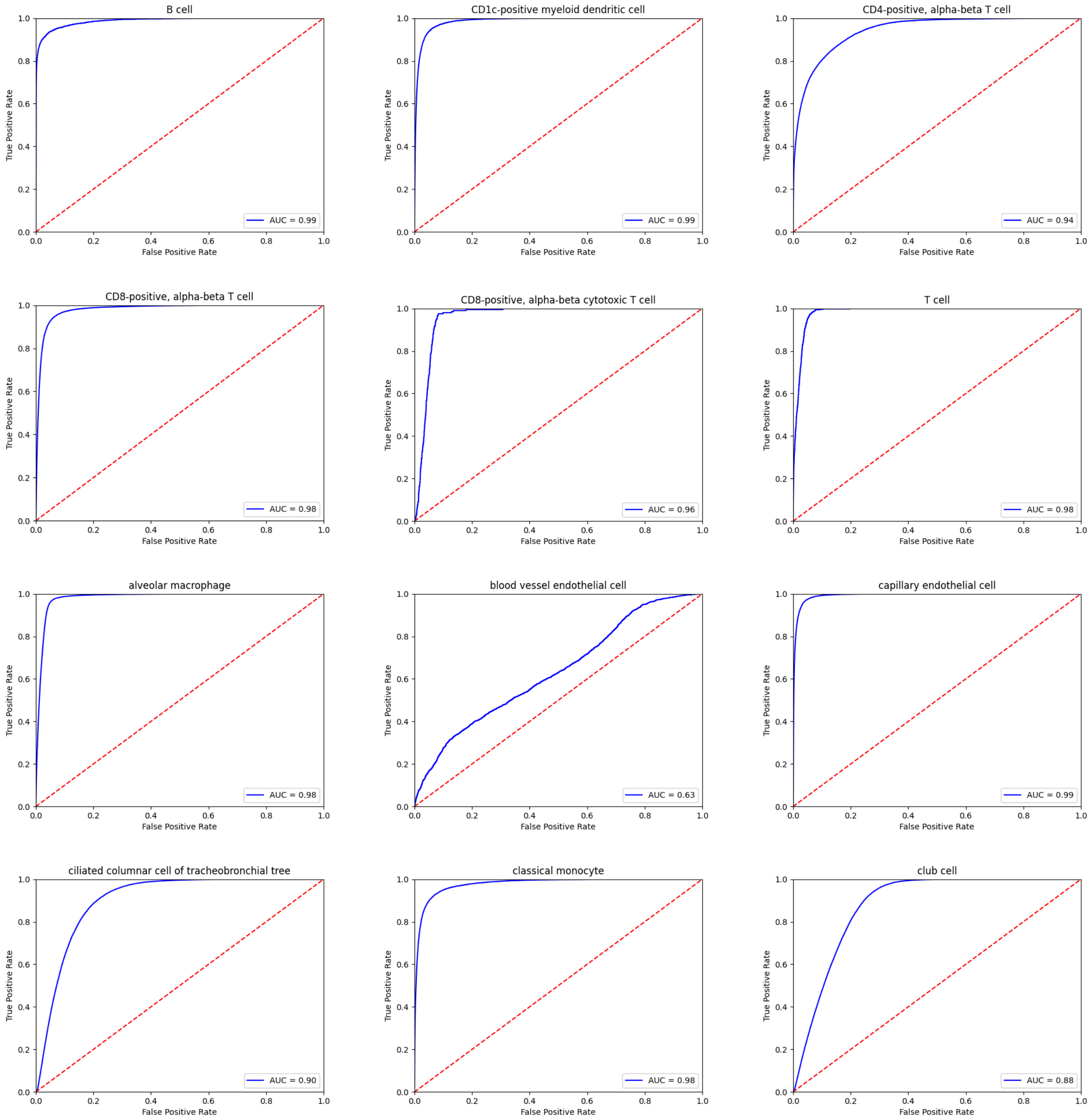

Figure S5. Example AUC for some cell types. The red line is the line for random predictions. The perfect prediction is when the line goes towards the upper left corner and the AUC is 1.00.

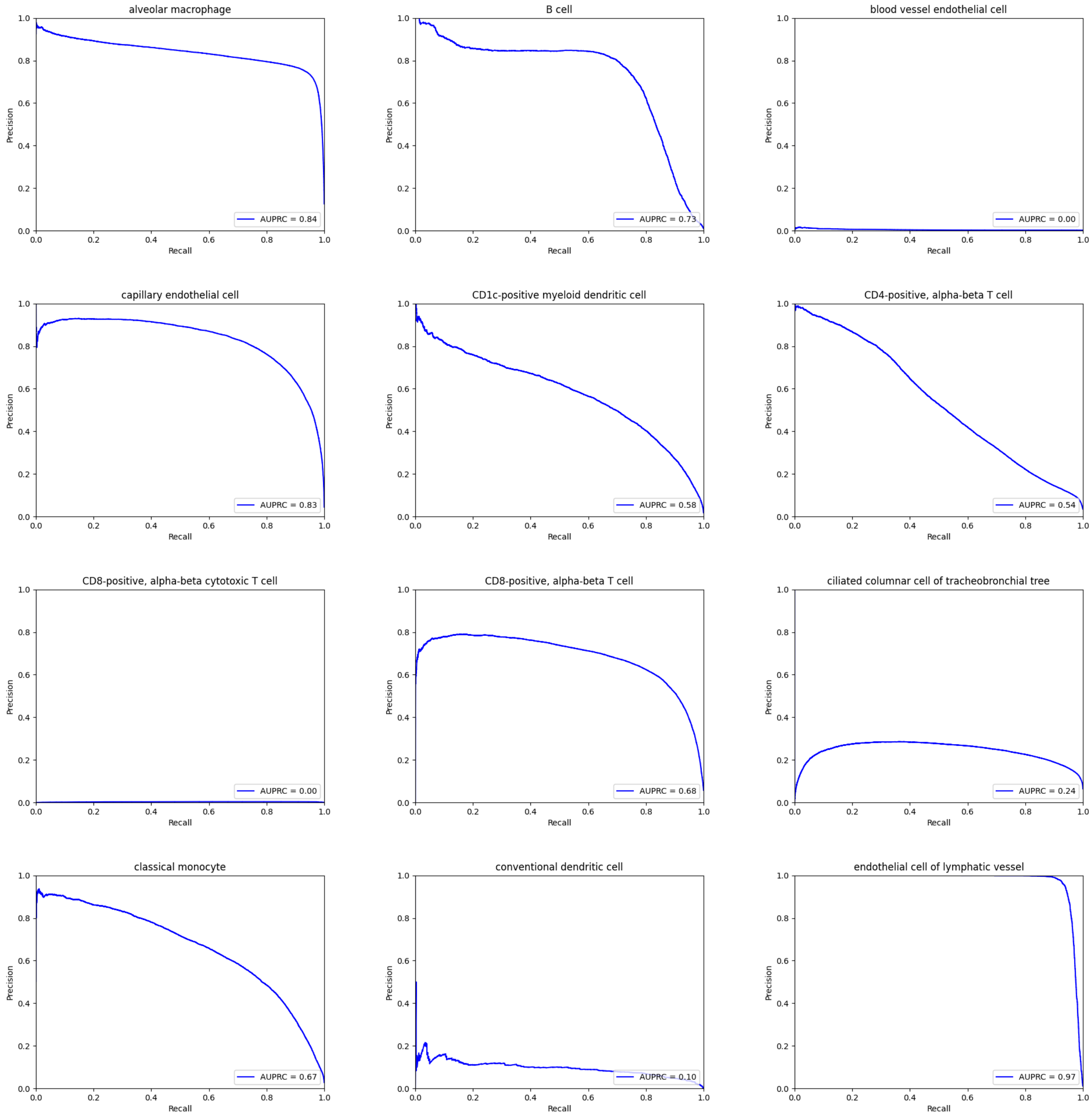

Figure S6. Precision-recall curve and AUPRC for example cell types. The perfect prediction is if the line goes towards the upper right corner and the AUPRC is 1.00.

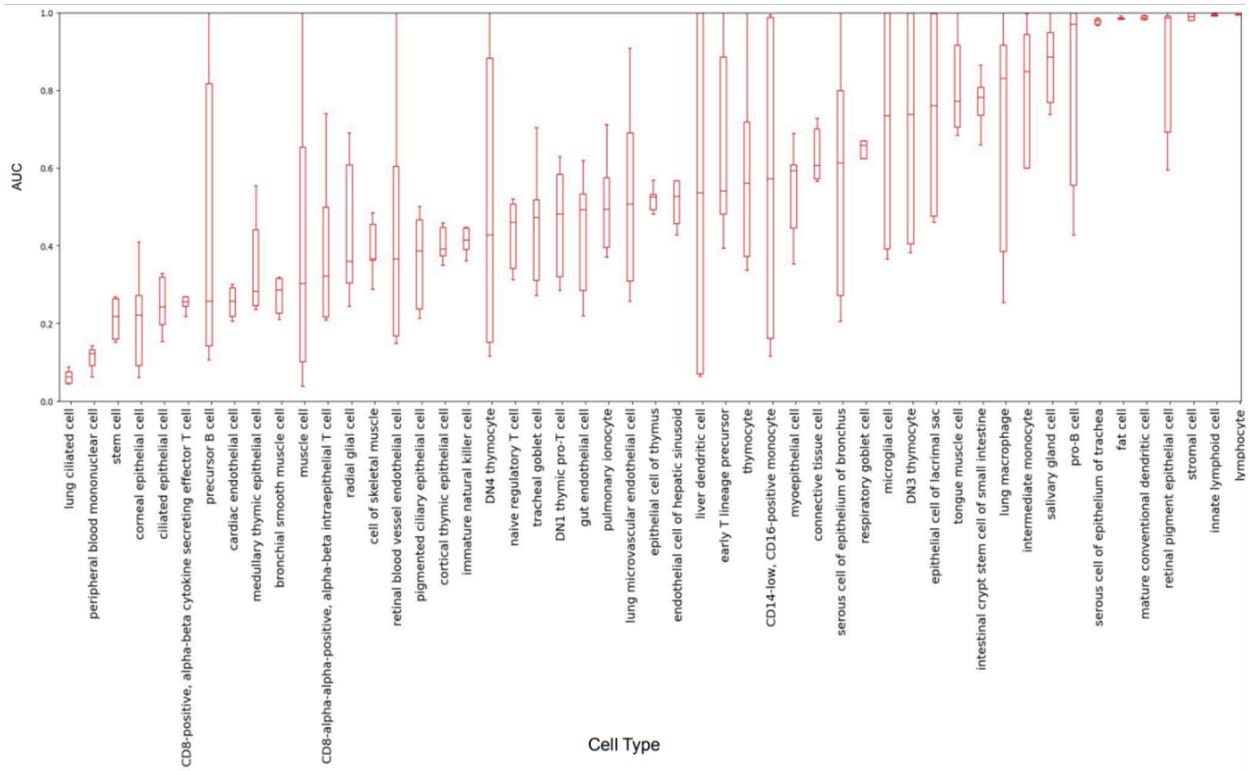

AUC
1.0
0.8
0.6
0.4
0.2
0.0
lung ciliated cell
peripheral blood mononuclear cell
stem cell
corneal epithelial cell
ciliated epithelial cell
CD8-positive, alpha-beta cytokine secreting effector T cell
precursor B cell
cardiac endothelial cell
medullary thymic epithelial cell
bronchial smooth muscle cell
muscle cell
CD8-alpha-alpha-positive, alpha-beta intraepithelial T cell
radial glial cell
cell of skeletal muscle
retinal blood vessel endothelial cell
pigmented ciliary epithelial cell
cortical thymic epithelial cell
immature natural killer cell
DN4 thymocyte
naive regulatory T cell
tracheal goblet cell
DN1 thymic pro-T cell
gut endothelial cell
pulmonary ionocyte
lung microvascular endothelial cell
epithelial cell of thymus
endothelial cell of hepatic sinusoid
liver dendritic cell
early T lineage precursor
thymocyte
CD14-low, CD16-positive monocyte
myoepithelial cell
connective tissue cell
serous cell of epithelium of bronchus
respiratory goblet cell
microglial cell
DN3 thymocyte
epithelial cell of lacrimal sac
tongue muscle cell
intestinal crypt stem cell of small intestine
lung macrophage
intermediate monocyte
salivary gland cell
pro-B cell
serous cell of epithelium of trachea
fat cell
mature conventional dendritic cell
retinal pigment epithelial cell
stromal cell
innate lymphoid cell
lymphocyte
Cell Type

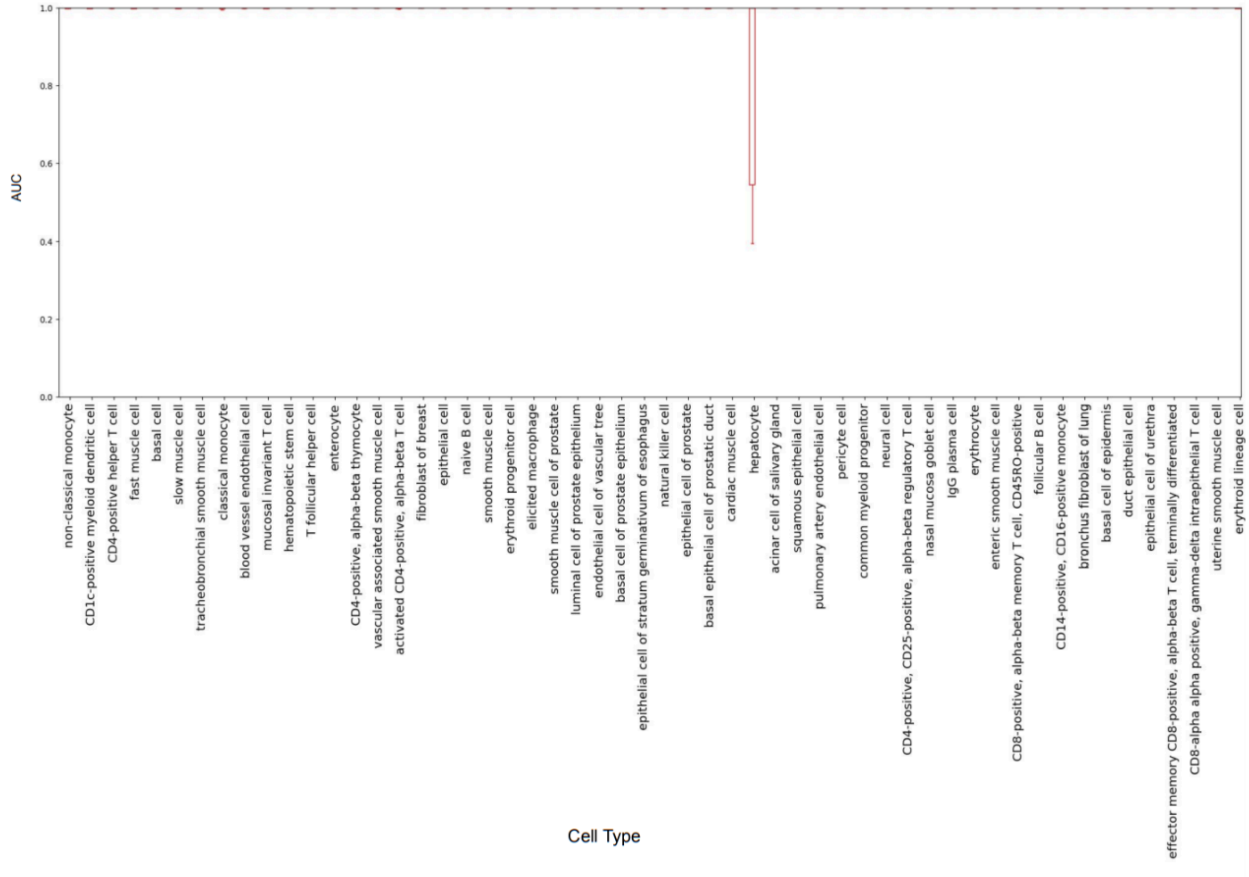

AUC
1.0
0.8
0.6
0.4
0.2
0.0
non-classical monocyte
CD1c-positive myeloid dendritic cell
CD4-positive helper T cell
fast muscle cell
basal cell
slow muscle cell
tracheobronchial smooth muscle cell
classical monocyte
blood vessel endothelial cell
mucosal invariant T cell
hematopoietic stem cell
T follicular helper cell
enterocyte
CD4-positive, alpha-beta thymocyte
vascular associated smooth muscle cell
activated CD4-positive, alpha-beta T cell
fibroblast of breast
epithelial cell
naive B cell
smooth muscle cell
erythroid progenitor cell
elicited macrophage
smooth muscle cell of prostate
luminal cell of prostate epithelium
endothelial cell of vascular tree
basal cell of prostate epithelium
epithelial cell of stratum germinativum of esophagus
natural killer cell
epithelial cell of prostate
basal epithelial cell of prostatic duct
cardiac muscle cell
hepatocyte
acinar cell of salivary gland
squamous epithelial cell
pulmonary artery endothelial cell
pericyte cell
common myeloid progenitor
neural cell
CD4-positive, CD25-positive, alpha-beta regulatory T cell
nasal mucosa goblet cell
IgG plasma cell
erythrocyte
enteric smooth muscle cell
CD8-positive, alpha-beta memory T cell, CD45RO-positive
follicular B cell
CD14-positive, CD16-positive monocyte
bronchus fibroblast of lung
basal cell of epidermis
duct epithelial cell
epithelial cell of urethra
effector memory CD8-positive, alpha-beta T cell, terminally differentiated
CD8-alpha alpha positive, gamma-delta intraepithelial T cell
uterine smooth muscle cell
erythroid lineage cell
Cell Type

Figure S7. AUC distribution of cross-cell evaluation. The plot included all cell types that appeared more than 4 times in the test set.

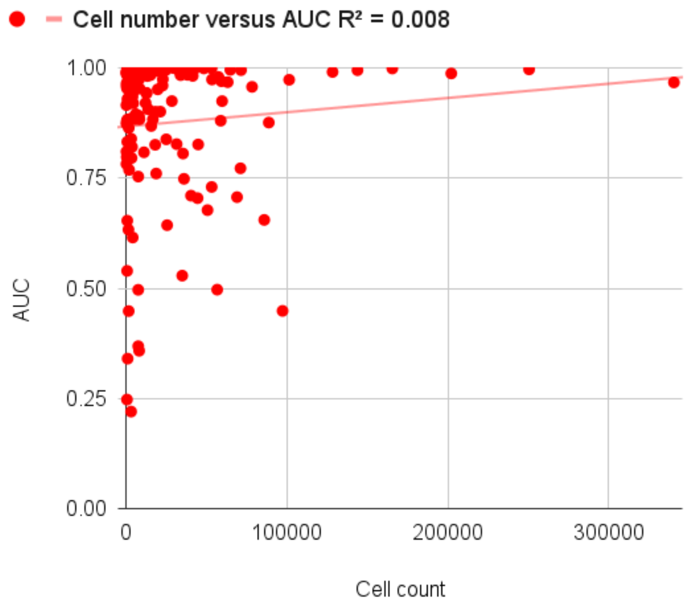

Figure S8. Performance in AUC versus cell counts of each cell type.

Table S1. Data sets used for training and evaluation (healthy human cells).

File Name	Data Set	Tissue		Assay	Cells
global.h5ad	Global	Cross-tissue immune cell analysis reveals tissue-specific features in humans	17 tissues	3 assays	329762
tabula_sapiens_stromal.h5ad	Tabula Sapiens - Stromal	Tabula Sapiens	39 tissues	10x 3' v3, Smart-seq2	82478
tabula_sapiens_endothelial.h5ad	Tabula Sapiens - Endothelial	Tabula Sapiens	40 tissues	10x 3' v3, Smart-seq2	31,691
tabula_sapiens_epithelial.h5ad	Tabula Sapiens - Epithelial	Tabula Sapiens	32 tissues	10x 3' v3, Smart-seq2	104148
t_innate_lymphoid.h5ad	T & innate lymphoid cells	Cross-tissue immune cell analysis reveals tissue-specific features in humans	17 tissues	3 assays	216611
tabula_sapiens_immune.h5ad	Tabula Sapiens - Immune	Tabula Sapiens	44 tissues	10x 3' v3, Smart-seq2	264824
singlecellblood1.h5ad	Single-cell eQTL mapping identifies cell type specific genetic control of autoimmune disease	Single-cell eQTL mapping identifies cell type specific genetic control of autoimmune disease	blood	10x 3' v2	1,248,980
human_lung_atlas.h5ad	The integrated Human Lung Cell Atlas	The integrated Human Lung Cell Atlas	3 tissues	5 assays	584884
non_pregnant_uterus.h5ad	Non-pregnant Uterus (Endometrium - All)	Mapping the temporal and spatial dynamics of the human endometrium in vivo and in vitro	uterus	10x 3' v2, 10x 3' v3	100307
human_retina.h5ad	All major cell types in adult human retina	Single-cell transcriptomic atlas for adult human retina	3 tissues	10x 3' v3	244474
all_cells_human_heart.h5ad	All — Cells of the adult human heart	Cells of the adult human heart	6 tissues	10x 3' v2, 10x 3' v3	486134
ventricular_cardiomyocytes.h5ad	Ventricular cardiomyocytes — Cells of the adult human heart	Cells of the adult human heart	4 tissues	10x 3' v2, 10x 3' v3	125289
vascular_human_heart.h5ad	Vascular — Cells of the adult human heart	Cells of the adult human heart	6 tissues	10x 3' v2, 10x 3' v3	195395
esophagus_epithelium.h5ad	Esophagus Epithelium	scRNA-seq assessment of the human lung, spleen, and esophagus tissue stability after cold preservation	epithelium of esophagus	10x 3' v2	87947
spleen.h5ad	Spleen	scRNA-seq assessment of the human lung, spleen, and esophagus tissue stability after cold preservation	spleen	10x 3' v2	94256

human_thymic.h5ad	Figure 1 - A cell atlas of human thymic development defines T cell repertoire formation	A cell atlas of human thymic development defines T cell repertoire formation	thymus	3 assays	255901
single_cell_analysis.h5ad	Single cell analysis of mouse and human prostate reveals novel fibroblasts with specialized distribution and microenvironment interactions - All Human Cells	Single cell analysis of mouse and human prostate reveals novel fibroblasts with specialized distribution and microenvironment interactions	peripheral zone of prostate, transition zone of prostate, benign prostatic hyperplasia	10x 3' v2, 10x 3' v3	83451

Table S2: Average AUC for each cell type.

endothelial cell of lymphatic vessel	0.9868089122	epithelial cell	0.7397730289
cardiac endothelial cell	0.2095881969	effector memory CD4-positive, alpha-beta T cell	0.7708770563
endothelial cell of vascular tree	0.5437332314	CD16-negative, CD56-bright natural killer cell, human	0.8364612824
blood vessel endothelial cell	0.7048978801	mucosal invariant T cell	0.7575844291
slow muscle cell	0.7500956742	gamma-delta T cell	0.5575306926
fast muscle cell	0.7974134363	alpha-beta T cell	0.701934634
dendritic cell	0.9160293482	group 3 innate lymphoid cell	0.7425098433
mast cell	0.9955482911	CD8-positive, alpha-beta memory T cell, CD45RO-positive	0.9992672546
macrophage	0.9508096241	effector memory CD8-positive, alpha-beta T cell, terminally differentiated	0.9991172442
Schwann cell	0.6368551293	CD16-positive, CD56-dim natural killer cell, human	0.9999067262
neutrophil	0.7198621846	regular ventricular cardiac myocyte	0.9945456481
T cell	0.821608155	basal cell of prostate epithelium	0.8162521446
mature NK T cell	0.9016708944	secretory cell	0.4285405757
luminal epithelial cell of mammary gland	0.8722352566	retinal bipolar neuron	0.2206481616
basal cell	0.7279305484	retina horizontal cell	0.492931926
glandular epithelial cell	0.701403236	mesothelial cell	0.9782187166
fibroblast	0.9585327053	ciliated cell	0.3419067542
fibroblast of cardiac tissue	0.4515998029	ionocyte	0.9874735095
B cell	0.9626824057	club cell	0.674173165
myeloid dendritic cell	0.8140439132	medullary thymic epithelial cell	0.2950644535
smooth muscle cell of prostate	0.9429398355	type II pneumocyte	0.987162743
cardiac muscle cell	0.4753875827	type I pneumocyte	0.887173021
smooth muscle cell	0.8284258903	ciliated columnar cell of tracheobronchial tree	0.7613831025
respiratory basal cell	0.6111392274	nasal mucosa goblet cell	0.6540197955
alveolar macrophage	0.9476721103	vein endothelial cell	0.9904531464
elicited macrophage	0.6671593978	capillary endothelial cell	0.9912067418
luminal cell of prostate epithelium	0.9852898835	plasma cell	0.9812073176
keratinocyte	0.5637771707	classical monocyte	0.967964569
monocyte	0.8882405253	non-classical monocyte	0.9567476048
CD8-positive, alpha-beta T cell	0.8973342838	CD1c-positive myeloid dendritic cell	0.9832576146
CD4-positive, alpha-beta T cell	0.9334215585	pericyte cell	0.9539106664

platelet	0.8373590119	myofibroblast cell	0.775291574
naive thymus-derived CD4-positive, alpha-beta T cell	0.8240613644	mesenchymal stem cell	0.5288356922
hematopoietic stem cell	0.8856913309	vascular associated smooth muscle cell	0.7627784595
plasmacytoid dendritic cell	0.9366703992	endothelial cell	0.7970325535
innate lymphoid cell	0.7498940833	endothelial cell of artery	0.9943848971
regulatory T cell	0.8127882553	erythrocyte	0.7570402217
CD4-positive helper T cell	0.7868717797	naive B cell	0.6810227534
CD8-positive, alpha-beta memory T cell	0.7967017047	memory B cell	0.8000734984
naive thymus-derived CD8-positive, alpha-beta T cell	0.5534900238	leukocyte	0.5419669875
CD8-positive, alpha-beta cytotoxic T cell	0.9290202526	erythroid lineage cell	0.9293223631
plasmablast	0.8510171139	myeloid cell	0.7460730371
T follicular helper cell	0.9399968255	double negative thymocyte	0.3165715633
natural killer cell	0.9346257784	precursor B cell	0.1698407516
activated CD8-positive, alpha-beta T cell	0.9719653112	progenitor cell	0.9025291138
lymphocyte	0.6813826032	megakaryocyte	0.7798800537
conventional dendritic cell	0.898674239	double-positive, alpha-beta thymocyte	0.3381953241
native cell	0.7698638981	CD4-positive, alpha-beta memory T cell	0.3961981113
stromal cell	0.8144876203	CD14-positive monocyte	0.8553260471
mucus secreting cell	0.8290732115	CD4-positive, alpha-beta cytotoxic T cell	0.7721295137

Table S3. The relationship of children-parent cell types.

acinar cell	glandular epithelial cell
acinar cell	epithelial cell
acinar cell	secretory cell
acinar cell of salivary gland	epithelial cell
acinar cell of salivary gland	glandular epithelial cell
acinar cell of salivary gland	acinar cell
acinar cell of salivary gland	secretory cell
activated CD4-positive, alpha-beta T cell	CD4-positive, alpha-beta T cell
activated CD4-positive, alpha-beta T cell	alpha-beta T cell
activated CD4-positive, alpha-beta T cell	T cell
activated CD4-positive, alpha-beta T cell	lymphocyte
activated CD4-positive, alpha-beta T cell	leukocyte
activated CD8-positive, alpha-beta T cell	lymphocyte
activated CD8-positive, alpha-beta T cell	leukocyte
activated CD8-positive, alpha-beta T cell	alpha-beta T cell
activated CD8-positive, alpha-beta T cell	T cell
activated CD8-positive, alpha-beta T cell	CD8-positive, alpha-beta T cell
adventitial cell	connective tissue cell
adventitial cell	supportive cell
alpha-beta T cell	T cell
alpha-beta T cell	lymphocyte
alpha-beta T cell	leukocyte
alpha-beta T cell	alpha-beta T cell
alveolar macrophage	leukocyte
alveolar macrophage	macrophage
alveolar macrophage	lung macrophage
alveolar macrophage	myeloid cell

alveolar macrophage	phagocyte
amacrine cell	neural cell
amacrine cell	neuron
animal cell	native cell
B cell	lymphocyte
B cell	leukocyte
basal cell	stem cell
basal cell of epidermis	epithelial cell
basal cell of epidermis	epidermal cell
basal cell of epidermis	keratinocyte
basal cell of epidermis	basal cell of epidermis
basal cell of epidermis	squamous epithelial cell
basal cell of epidermis	stratified epithelial cell
basal cell of epidermis	stem cell
basal cell of epidermis	basal cell
basal cell of epidermis	progenitor cell
basal cell of prostate epithelium	epithelial cell of prostate
basal cell of prostate epithelium	epithelial cell
basal epithelial cell of prostatic duct	duct epithelial cell
basal epithelial cell of prostatic duct	epithelial cell
basal epithelial cell of prostatic duct	epithelial cell of prostate
basal epithelial cell of prostatic duct	basal cell of prostate epithelium
basophil	granulocyte
basophil	leukocyte
basophil	myeloid cell
bladder urothelial cell	epithelial cell
blood vessel endothelial cell	endothelial cell of vascular tree
blood vessel endothelial cell	endothelial cell

blood vessel endothelial cell	epithelial cell
blood vessel endothelial cell	squamous epithelial cell
bronchial goblet cell	epithelial cell
bronchial goblet cell	tracheobronchial goblet cell
bronchial goblet cell	glandular epithelial cell
bronchial goblet cell	goblet cell
bronchial goblet cell	respiratory goblet cell
bronchial goblet cell	secretory cell
bronchial goblet cell	mucus secreting cell
bronchial smooth muscle cell	smooth muscle cell
bronchial smooth muscle cell	muscle cell
bronchial smooth muscle cell	tracheobronchial smooth muscle cell
bronchial smooth muscle cell	contractile cell
bronchus fibroblast of lung	fibroblast of lung
bronchus fibroblast of lung	fibroblast
bronchus fibroblast of lung	connective tissue cell
brush cell of trachebronchial tree	epithelial cell
capillary endothelial cell	blood vessel endothelial cell
capillary endothelial cell	endothelial cell of vascular tree
capillary endothelial cell	endothelial cell
capillary endothelial cell	epithelial cell
capillary endothelial cell	squamous epithelial cell
cardiac endothelial cell	epithelial cell
cardiac endothelial cell	endothelial cell
cardiac muscle cell	muscle cell
cardiac muscle cell	contractile cell
CD14-low, CD16-positive monocyte	CD14-positive monocyte
CD14-low, CD16-positive monocyte	monocyte

CD14-low, CD16-positive monocyte	leukocyte
CD14-low, CD16-positive monocyte	non-classical monocyte
CD14-low, CD16-positive monocyte	myeloid cell
CD14-low, CD16-positive monocyte	progenitor cell
CD14-positive monocyte	monocyte
CD14-positive monocyte	leukocyte
CD14-positive monocyte	myeloid cell
CD14-positive monocyte	progenitor cell
CD14-positive, CD16-positive monocyte	CD14-positive monocyte
CD14-positive, CD16-positive monocyte	monocyte
CD14-positive, CD16-positive monocyte	leukocyte
CD14-positive, CD16-positive monocyte	myeloid cell
CD14-positive, CD16-positive monocyte	progenitor cell
CD141-positive myeloid dendritic cell	myeloid dendritic cell
CD141-positive myeloid dendritic cell	conventional dendritic cell
CD141-positive myeloid dendritic cell	dendritic cell
CD141-positive myeloid dendritic cell	leukocyte
CD141-positive myeloid dendritic cell	plasmacytoid dendritic cell
CD141-positive myeloid dendritic cell	professional antigen presenting cell
CD141-positive myeloid dendritic cell	myeloid cell
CD16-negative, CD56-bright natural killer cell, human	natural killer cell
CD16-negative, CD56-bright natural killer cell, human	innate lymphoid cell
CD16-negative, CD56-bright natural killer cell, human	lymphocyte
CD16-negative, CD56-bright natural killer cell, human	leukocyte
CD16-positive, CD56-dim natural killer cell, human	natural killer cell
CD16-positive, CD56-dim natural killer cell, human	innate lymphoid cell
CD16-positive, CD56-dim natural killer cell, human	natural killer cell
CD16-positive, CD56-dim natural killer cell, human	lymphocyte

CD16-positive, CD56-dim natural killer cell, human	leukocyte
CD1c-positive myeloid dendritic cell	myeloid dendritic cell
CD1c-positive myeloid dendritic cell	conventional dendritic cell
CD1c-positive myeloid dendritic cell	dendritic cell
CD1c-positive myeloid dendritic cell	leukocyte
CD1c-positive myeloid dendritic cell	professional antigen presenting cell
CD1c-positive myeloid dendritic cell	myeloid cell
CD4-positive helper T cell	CD4-positive, alpha-beta T cell
CD4-positive helper T cell	alpha-beta T cell
CD4-positive helper T cell	T cell
CD4-positive helper T cell	lymphocyte
CD4-positive helper T cell	leukocyte
CD4-positive, alpha-beta cytotoxic T cell	CD4-positive, alpha-beta T cell
CD4-positive, alpha-beta cytotoxic T cell	alpha-beta T cell
CD4-positive, alpha-beta cytotoxic T cell	T cell
CD4-positive, alpha-beta cytotoxic T cell	lymphocyte
CD4-positive, alpha-beta cytotoxic T cell	leukocyte
CD4-positive, alpha-beta memory T cell	alpha-beta T cell
CD4-positive, alpha-beta memory T cell	T cell
CD4-positive, alpha-beta memory T cell	lymphocyte
CD4-positive, alpha-beta memory T cell	leukocyte
CD4-positive, alpha-beta T cell	alpha-beta T cell
CD4-positive, alpha-beta T cell	T cell
CD4-positive, alpha-beta T cell	lymphocyte
CD4-positive, alpha-beta T cell	leukocyte
CD4-positive, alpha-beta thymocyte	alpha-beta T cell
CD4-positive, alpha-beta thymocyte	T cell
CD4-positive, alpha-beta thymocyte	lymphocyte

CD4-positive, alpha-beta thymocyte	leukocyte
CD4-positive, alpha-beta thymocyte	thymocyte
CD4-positive, CD25-positive, alpha-beta regulatory T cell	CD4-positive, alpha-beta T cell
CD4-positive, CD25-positive, alpha-beta regulatory T cell	alpha-beta T cell
CD4-positive, CD25-positive, alpha-beta regulatory T cell	T cell
CD4-positive, CD25-positive, alpha-beta regulatory T cell	lymphocyte
CD4-positive, CD25-positive, alpha-beta regulatory T cell	leukocyte
CD4-positive, CD25-positive, alpha-beta regulatory T cell	regulatory T cell
CD8-alpha alpha positive, gamma-delta intraepithelial T cell	gamma-delta T cell
CD8-alpha alpha positive, gamma-delta intraepithelial T cell	T cell
CD8-alpha alpha positive, gamma-delta intraepithelial T cell	lymphocyte
CD8-alpha alpha positive, gamma-delta intraepithelial T cell	leukocyte
CD8-alpha-alpha-positive, alpha-beta intraepithelial T cell	alpha-beta T cell
CD8-alpha-alpha-positive, alpha-beta intraepithelial T cell	T cell
CD8-alpha-alpha-positive, alpha-beta intraepithelial T cell	lymphocyte
CD8-alpha-alpha-positive, alpha-beta intraepithelial T cell	leukocyte
CD8-positive, alpha-beta cytokine secreting effector T cell	CD8-positive, alpha-beta T cell
CD8-positive, alpha-beta cytokine secreting effector T cell	alpha-beta T cell
CD8-positive, alpha-beta cytokine secreting effector T cell	T cell
CD8-positive, alpha-beta cytokine secreting effector T cell	lymphocyte
CD8-positive, alpha-beta cytokine secreting effector T cell	leukocyte
CD8-positive, alpha-beta cytotoxic T cell	CD8-positive, alpha-beta T cell
CD8-positive, alpha-beta cytotoxic T cell	alpha-beta T cell
CD8-positive, alpha-beta cytotoxic T cell	T cell
CD8-positive, alpha-beta cytotoxic T cell	lymphocyte
CD8-positive, alpha-beta cytotoxic T cell	leukocyte
CD8-positive, alpha-beta memory T cell	CD8-positive, alpha-beta T cell
CD8-positive, alpha-beta memory T cell	alpha-beta T cell

CD8-positive, alpha-beta memory T cell	T cell
CD8-positive, alpha-beta memory T cell	lymphocyte
CD8-positive, alpha-beta memory T cell	leukocyte
CD8-positive, alpha-beta memory T cell, CD45RO-positive	CD8-positive, alpha-beta memory T cell
CD8-positive, alpha-beta memory T cell, CD45RO-positive	CD8-positive, alpha-beta T cell
CD8-positive, alpha-beta memory T cell, CD45RO-positive	alpha-beta T cell
CD8-positive, alpha-beta memory T cell, CD45RO-positive	T cell
CD8-positive, alpha-beta memory T cell, CD45RO-positive	lymphocyte
CD8-positive, alpha-beta memory T cell, CD45RO-positive	leukocyte
CD8-positive, alpha-beta T cell	alpha-beta T cell
CD8-positive, alpha-beta T cell	T cell
CD8-positive, alpha-beta T cell	lymphocyte
CD8-positive, alpha-beta T cell	leukocyte
CD8-positive, alpha-beta thymocyte	alpha-beta T cell
CD8-positive, alpha-beta thymocyte	T cell
CD8-positive, alpha-beta thymocyte	lymphocyte
CD8-positive, alpha-beta thymocyte	leukocyte
CD8-positive, alpha-beta thymocyte	thymocyte
central memory CD4-positive, alpha-beta T cell	CD4-positive, alpha-beta memory T cell
central memory CD4-positive, alpha-beta T cell	CD4-positive, alpha-beta T cell
central memory CD4-positive, alpha-beta T cell	alpha-beta T cell
central memory CD4-positive, alpha-beta T cell	T cell
central memory CD4-positive, alpha-beta T cell	lymphocyte
central memory CD4-positive, alpha-beta T cell	leukocyte
central memory CD8-positive, alpha-beta T cell	CD8-positive, alpha-beta memory T cell, CD45RO-positive
central memory CD8-positive, alpha-beta T cell	CD8-positive, alpha-beta memory T cell
central memory CD8-positive, alpha-beta T cell	CD8-positive, alpha-beta T cell

central memory CD8-positive, alpha-beta T cell	alpha-beta T cell
central memory CD8-positive, alpha-beta T cell	T cell
central memory CD8-positive, alpha-beta T cell	lymphocyte
central memory CD8-positive, alpha-beta T cell	leukocyte
ciliated columnar cell of tracheobronchial tree	multi-ciliated epithelial cell
ciliated columnar cell of tracheobronchial tree	ciliated epithelial cell
ciliated columnar cell of tracheobronchial tree	epithelial cell
ciliated columnar cell of tracheobronchial tree	ciliated cell
ciliated epithelial cell	epithelial cell
ciliated epithelial cell	ciliated cell
classical monocyte	monocyte
classical monocyte	leukocyte
classical monocyte	myeloid cell
classical monocyte	phagocyte
classical monocyte	progenitor cell
club cell	epithelial cell
club cell	secretory cell
common myeloid progenitor	hematopoietic precursor cell
conjunctival epithelial cell	epithelial cell
conventional dendritic cell	dendritic cell
conventional dendritic cell	leukocyte
conventional dendritic cell	professional antigen presenting cell
corneal epithelial cell	epithelial cell
corneal epithelial cell	squamous epithelial cell
cortical thymic epithelial cell	epithelial cell
cortical thymic epithelial cell	epithelial cell of thymus
dendritic cell	leukocyte
dendritic cell	professional antigen presenting cell

dendritic cell, human	dendritic cell
dendritic cell, human	leukocyte
dendritic cell, human	professional antigen presenting cell
DN1 thymic pro-T cell	hematopoietic precursor cell
DN1 thymic pro-T cell	progenitor cell
DN3 thymocyte	thymocyte
DN3 thymocyte	T cell
DN3 thymocyte	lymphocyte
DN3 thymocyte	leukocyte
DN3 thymocyte	double negative thymocyte
DN4 thymocyte	thymocyte
DN4 thymocyte	T cell
DN4 thymocyte	lymphocyte
DN4 thymocyte	leukocyte
DN4 thymocyte	double negative thymocyte
double negative thymocyte	thymocyte
double negative thymocyte	T cell
double negative thymocyte	lymphocyte
double negative thymocyte	leukocyte
double-positive, alpha-beta thymocyte	
double-positive, alpha-beta thymocyte	alpha-beta T cell
double-positive, alpha-beta thymocyte	T cell
double-positive, alpha-beta thymocyte	lymphocyte
double-positive, alpha-beta thymocyte	leukocyte
double-positive, alpha-beta thymocyte	thymocyte
duct epithelial cell	epithelial cell
duodenum glandular cell	epithelial cell
duodenum glandular cell	glandular epithelial cell

duodenum glandular cell	secretory cell
early T lineage precursor	progenitor cell
effector CD4-positive, alpha-beta T cell	CD4-positive, alpha-beta T cell
effector CD4-positive, alpha-beta T cell	alpha-beta T cell
effector CD4-positive, alpha-beta T cell	T cell
effector CD4-positive, alpha-beta T cell	lymphocyte
effector CD4-positive, alpha-beta T cell	leukocyte
effector CD8-positive, alpha-beta T cell	CD8-positive, alpha-beta T cell
effector CD8-positive, alpha-beta T cell	alpha-beta T cell
effector CD8-positive, alpha-beta T cell	T cell
effector CD8-positive, alpha-beta T cell	lymphocyte
effector CD8-positive, alpha-beta T cell	leukocyte
effector memory CD4-positive, alpha-beta T cell	CD4-positive, alpha-beta memory T cell
effector memory CD4-positive, alpha-beta T cell	CD4-positive, alpha-beta T cell
effector memory CD4-positive, alpha-beta T cell	alpha-beta T cell
effector memory CD4-positive, alpha-beta T cell	T cell
effector memory CD4-positive, alpha-beta T cell	lymphocyte
effector memory CD4-positive, alpha-beta T cell	leukocyte
effector memory CD8-positive, alpha-beta T cell	CD8-positive, alpha-beta memory T cell, CD45RO-positive
effector memory CD8-positive, alpha-beta T cell	CD8-positive, alpha-beta memory T cell
effector memory CD8-positive, alpha-beta T cell	CD8-positive, alpha-beta T cell
effector memory CD8-positive, alpha-beta T cell	alpha-beta T cell
effector memory CD8-positive, alpha-beta T cell	T cell
effector memory CD8-positive, alpha-beta T cell	lymphocyte
effector memory CD8-positive, alpha-beta T cell	leukocyte
effector memory CD8-positive, alpha-beta T cell, terminally differentiated	CD8-positive, alpha-beta memory T cell

effector memory CD8-positive, alpha-beta T cell, terminally differentiated	CD8-positive, alpha-beta T cell
effector memory CD8-positive, alpha-beta T cell, terminally differentiated	alpha-beta T cell
effector memory CD8-positive, alpha-beta T cell, terminally differentiated	T cell
effector memory CD8-positive, alpha-beta T cell, terminally differentiated	lymphocyte
effector memory CD8-positive, alpha-beta T cell, terminally differentiated	leukocyte
elicited macrophage	macrophage
elicited macrophage	leukocyte
elicited macrophage	professional antigen presenting cell
elicited macrophage	myeloid cell
elicited macrophage	phagocyte
endothelial cell	epithelial cell
endothelial cell of artery	endothelial cell
endothelial cell of artery	epithelial cell
endothelial cell of artery	endothelial cell of vascular tree
endothelial cell of artery	blood vessel endothelial cell
endothelial cell of artery	squamous epithelial cell
endothelial cell of hepatic sinusoid	endothelial cell
endothelial cell of hepatic sinusoid	epithelial cell
endothelial cell of lymphatic vessel	endothelial cell of vascular tree
endothelial cell of lymphatic vessel	endothelial cell
endothelial cell of lymphatic vessel	epithelial cell
endothelial cell of vascular tree	endothelial cell
endothelial cell of vascular tree	epithelial cell
enteric smooth muscle cell	smooth muscle cell
enteric smooth muscle cell	muscle cell

enteric smooth muscle cell	contractile cell
enterocyte	epithelial cell
enterocyte	ciliated epithelial cell
enterocyte	ciliated cell
enterocyte of epithelium of large intestine	enterocyte
enterocyte of epithelium of large intestine	epithelial cell
enterocyte of epithelium of large intestine	ciliated epithelial cell
enterocyte of epithelium of large intestine	ciliated cell
enterocyte of epithelium of small intestine	enterocyte
enterocyte of epithelium of small intestine	epithelial cell
enterocyte of epithelium of small intestine	ciliated epithelial cell
enterocyte of epithelium of small intestine	ciliated cell
epicardial adipocyte	connective tissue cell
epicardial adipocyte	fat cell
epidermal cell	epithelial cell
epidermal Langerhans cell	conventional dendritic cell
epidermal Langerhans cell	dendritic cell
epidermal Langerhans cell	leukocyte
epidermal Langerhans cell	Langerhans cell
epidermal Langerhans cell	professional antigen presenting cell
epidermal Langerhans cell	myeloid cell
epithelial cell of alveolus of lung	epithelial cell
epithelial cell of alveolus of lung	epithelial cell of lung
epithelial cell of lacrimal sac	duct epithelial cell
epithelial cell of lacrimal sac	epithelial cell
epithelial cell of lung	epithelial cell
epithelial cell of prostate	epithelial cell
epithelial cell of stratum germinativum of esophagus	basal cell

epithelial cell of stratum germinativum of esophagus	stem cell
epithelial cell of sweat gland	epidermal cell
epithelial cell of sweat gland	epithelial cell
epithelial cell of thymus	epithelial cell
epithelial cell of urethra	epithelial cell
epithelial cell of uterus	epithelial cell
erythrocyte	myeloid cell
erythrocyte	erythroid lineage cell
erythroid lineage cell	myeloid cell
erythroid progenitor cell	hematopoietic precursor cell
erythroid progenitor cell	myeloid cell
erythroid progenitor cell	erythroid lineage cell
erythroid progenitor cell	progenitor cell
eye photoreceptor cell	neuron
eye photoreceptor cell	neural cell
fast muscle cell	skeletal muscle fiber
fast muscle cell	muscle cell
fast muscle cell	contractile cell
fast muscle cell	cell of skeletal muscle
fat cell	connective tissue cell
fibroblast	connective tissue cell
fibroblast of breast	fibroblast
fibroblast of breast	connective tissue cell
fibroblast of cardiac tissue	fibroblast
fibroblast of cardiac tissue	connective tissue cell
fibroblast of connective tissue of glandular part of prostate	fibroblast
fibroblast of connective tissue of glandular part of prostate	connective tissue cell
fibroblast of connective tissue of nonglandular part of prostate	fibroblast

fibroblast of connective tissue of nonglandular part of prostate	connective tissue cell
fibroblast of lung	fibroblast
fibroblast of lung	connective tissue cell
follicular B cell	B cell
follicular B cell	lymphocyte
follicular B cell	leukocyte
follicular B cell	professional antigen presenting cell
gamma-delta T cell	T cell
gamma-delta T cell	lymphocyte
gamma-delta T cell	leukocyte
germinal center B cell	B cell
germinal center B cell	lymphocyte
germinal center B cell	leukocyte
germinal center B cell	professional antigen presenting cell
glandular epithelial cell	epithelial cell
glandular epithelial cell	secretory cell
glial cell	neural cell
goblet cell	glandular epithelial cell
goblet cell	epithelial cell
goblet cell	secretory cell
goblet cell	mucus secreting cell
granulocyte	leukocyte
granulocyte	myeloid cell
group 3 innate lymphoid cell	innate lymphoid cell
group 3 innate lymphoid cell	lymphocyte
group 3 innate lymphoid cell	leukocyte
gut endothelial cell	endothelial cell
gut endothelial cell	epithelial cell

hematopoietic stem cell	hematopoietic precursor cell
hematopoietic stem cell	stem cell
hematopoietic stem cell	progenitor cell
hepatocyte	epithelial cell
IgG plasma cell	plasma cell
IgG plasma cell	lymphocyte
IgG plasma cell	leukocyte
IgM plasma cell	plasma cell
IgM plasma cell	lymphocyte
IgM plasma cell	leukocyte
immature B cell	B cell
immature B cell	lymphocyte
immature B cell	leukocyte
immature natural killer cell	natural killer cell
immature natural killer cell	innate lymphoid cell
immature natural killer cell	lymphocyte
immature natural killer cell	leukocyte
innate lymphoid cell	lymphocyte
innate lymphoid cell	leukocyte
intermediate monocyte	monocyte
intermediate monocyte	leukocyte
intermediate monocyte	myeloid cell
intermediate monocyte	progenitor cell
interstitial cell of Cajal	epithelial cell
intestinal crypt stem cell	epithelial cell
intestinal crypt stem cell	stem cell
intestinal crypt stem cell of large intestine	epithelial cell
intestinal crypt stem cell of large intestine	intestinal crypt stem cell

intestinal crypt stem cell of large intestine	stem cell
intestinal crypt stem cell of small intestine	epithelial cell
intestinal crypt stem cell of small intestine	intestinal crypt stem cell
intestinal crypt stem cell of small intestine	stem cell
intestinal enteroendocrine cell	epithelial cell
intestinal enteroendocrine cell	glandular epithelial cell
intestinal enteroendocrine cell	secretory cell
intestinal tuft cell	epithelial cell
intrahepatic cholangiocyte	duct epithelial cell
intrahepatic cholangiocyte	epithelial cell
ionocyte	epithelial cell
keratinocyte	epidermal cell
keratinocyte	epithelial cell
keratinocyte	squamous epithelial cell
keratinocyte	stratified epithelial cell
keratocyte	fibroblast
keratocyte	connective tissue cell
kidney epithelial cell	epithelial cell
Langerhans cell	conventional dendritic cell
Langerhans cell	dendritic cell
Langerhans cell	leukocyte
Langerhans cell	professional antigen presenting cell
Langerhans cell	myeloid cell
large intestine goblet cell	epithelial cell
large intestine goblet cell	glandular epithelial cell
large intestine goblet cell	goblet cell
large intestine goblet cell	secretory cell
large intestine goblet cell	mucus secreting cell

liver dendritic cell	dendritic cell
liver dendritic cell	leukocyte
liver dendritic cell	professional antigen presenting cell
luminal cell of prostate epithelium	epithelial cell of prostate
luminal cell of prostate epithelium	epithelial cell
luminal epithelial cell of mammary gland	duct epithelial cell
luminal epithelial cell of mammary gland	epithelial cell
lung ciliated cell	ciliated cell
lung macrophage	macrophage
lung macrophage	leukocyte
lung macrophage	professional antigen presenting cell
lung macrophage	myeloid cell
lung macrophage	phagocyte
lung microvascular endothelial cell	epithelial cell of lung
lung microvascular endothelial cell	epithelial cell
lung microvascular endothelial cell	endothelial cell
lung microvascular endothelial cell	endothelial cell of vascular tree
lung microvascular endothelial cell	blood vessel endothelial cell
lung microvascular endothelial cell	squamous epithelial cell
lung neuroendocrine cell	neuroendocrine cell
lung neuroendocrine cell	epithelial cell
lung neuroendocrine cell	epithelial cell of lung
lung neuroendocrine cell	secretory cell
lymphocyte	leukocyte
macrophage	leukocyte
macrophage	professional antigen presenting cell
macrophage	myeloid cell
macrophage	phagocyte

mast cell	leukocyte
mast cell	myeloid cell
mast cell	secretory cell
mature conventional dendritic cell	conventional dendritic cell
mature conventional dendritic cell	dendritic cell
mature conventional dendritic cell	leukocyte
mature conventional dendritic cell	professional antigen presenting cell
mature conventional dendritic cell	myeloid cell
mature NK T cell	alpha-beta T cell
mature NK T cell	T cell
mature NK T cell	lymphocyte
mature NK T cell	leukocyte
medullary thymic epithelial cell	epithelial cell of thymus
medullary thymic epithelial cell	epithelial cell
megakaryocyte	myeloid cell
melanocyte of skin	melanocyte
memory B cell	B cell
memory B cell	lymphocyte
memory B cell	leukocyte
memory B cell	professional antigen presenting cell
mesenchymal stem cell	connective tissue cell
mesenchymal stem cell	stem cell
mesothelial cell	epithelial cell
mesothelial cell	squamous epithelial cell
microglial cell	macrophage
microglial cell	leukocyte
microglial cell	professional antigen presenting cell
microglial cell	myeloid cell

microglial cell	neural cell
microglial cell	glial cell
microglial cell	phagocyte
monocyte	leukocyte
monocyte	myeloid cell
monocyte	progenitor cell
mucosal invariant T cell	alpha-beta T cell
mucosal invariant T cell	T cell
mucosal invariant T cell	lymphocyte
mucosal invariant T cell	leukocyte
mucus secreting cell	secretory cell
Muller cell	radial glial cell
Muller cell	glial cell
Muller cell	neural cell
multi-ciliated epithelial cell	ciliated epithelial cell
multi-ciliated epithelial cell	epithelial cell
multi-ciliated epithelial cell	ciliated cell
muscle cell	contractile cell
myeloid dendritic cell	conventional dendritic cell
myeloid dendritic cell	dendritic cell
myeloid dendritic cell	leukocyte
myeloid dendritic cell	professional antigen presenting cell
myeloid dendritic cell	myeloid cell
myoepithelial cell	epithelial cell
myoepithelial cell	contractile cell
myoepithelial cell of mammary gland	myoepithelial cell
myoepithelial cell of mammary gland	myeloid cell
myoepithelial cell of mammary gland	epithelial cell

myoepithelial cell of mammary gland	contractile cell
myofibroblast cell	contractile cell
myometrial cell	smooth muscle cell
myometrial cell	muscle cell
myometrial cell	uterine smooth muscle cell
myometrial cell	contractile cell
naive B cell	B cell
naive B cell	lymphocyte
naive B cell	leukocyte
naive B cell	professional antigen presenting cell
naive regulatory T cell	CD4-positive, CD25-positive, alpha-beta regulatory T cell
naive regulatory T cell	CD4-positive, alpha-beta T cell
naive regulatory T cell	alpha-beta T cell
naive regulatory T cell	T cell
naive regulatory T cell	lymphocyte
naive regulatory T cell	leukocyte
naive regulatory T cell	regulatory T cell
naive thymus-derived CD4-positive, alpha-beta T cell	CD4-positive, alpha-beta T cell
naive thymus-derived CD4-positive, alpha-beta T cell	alpha-beta T cell
naive thymus-derived CD4-positive, alpha-beta T cell	T cell
naive thymus-derived CD4-positive, alpha-beta T cell	lymphocyte
naive thymus-derived CD4-positive, alpha-beta T cell	leukocyte
naive thymus-derived CD8-positive, alpha-beta T cell	CD8-positive, alpha-beta T cell
naive thymus-derived CD8-positive, alpha-beta T cell	alpha-beta T cell
naive thymus-derived CD8-positive, alpha-beta T cell	T cell
naive thymus-derived CD8-positive, alpha-beta T cell	lymphocyte
naive thymus-derived CD8-positive, alpha-beta T cell	leukocyte

nasal mucosa goblet cell	goblet cell
nasal mucosa goblet cell	glandular epithelial cell
nasal mucosa goblet cell	epithelial cell
nasal mucosa goblet cell	respiratory goblet cell
nasal mucosa goblet cell	secretory cell
nasal mucosa goblet cell	mucus secreting cell
natural killer cell	innate lymphoid cell
natural killer cell	lymphocyte
natural killer cell	leukocyte
neuroendocrine cell	epithelial cell
neuroendocrine cell	secretory cell
neuron	neural cell
neutrophil	granulocyte
neutrophil	leukocyte
neutrophil	myeloid cell
non-classical monocyte	monocyte
non-classical monocyte	leukocyte
non-classical monocyte	myeloid cell
non-classical monocyte	progenitor cell
pancreatic A cell	epithelial cell
pancreatic A cell	glandular epithelial cell
pancreatic A cell	secretory cell
pancreatic acinar cell	epithelial cell
pancreatic acinar cell	glandular epithelial cell
pancreatic acinar cell	acinar cell
pancreatic acinar cell	secretory cell
pancreatic D cell	epithelial cell
pancreatic D cell	glandular epithelial cell

pancreatic D cell	secretory cell
pancreatic ductal cell	duct epithelial cell
pancreatic ductal cell	epithelial cell
pancreatic PP cell	duct epithelial cell
pancreatic PP cell	epithelial cell
pancreatic PP cell	secretory cell
pancreatic stellate cell	fibroblast
pancreatic stellate cell	connective tissue cell
paneth cell of colon	epithelial cell
paneth cell of colon	glandular epithelial cell
paneth cell of colon	secretory cell
paneth cell of epithelium of small intestine	epithelial cell
paneth cell of epithelium of small intestine	glandular epithelial cell
paneth cell of epithelium of small intestine	secretory cell
peripheral blood mononuclear cell	leukocyte
phagocyte	professional antigen presenting cell
pigmented ciliary epithelial cell	multi-ciliated epithelial cell
pigmented ciliary epithelial cell	ciliated epithelial cell
pigmented ciliary epithelial cell	epithelial cell
pigmented ciliary epithelial cell	ciliated cell
plasma cell	lymphocyte
plasmablast	B cell
plasmablast	lymphocyte
plasmablast	leukocyte
plasmablast	professional antigen presenting cell
plasmacytoid dendritic cell	dendritic cell
plasmacytoid dendritic cell	leukocyte
plasmacytoid dendritic cell	professional antigen presenting cell

platelet	myeloid cell
platelet	secretory cell
precursor B cell	B cell
precursor B cell	lymphocyte
precursor B cell	leukocyte
pro-B cell	hematopoietic precursor cell
pro-B cell	progenitor cell
professional antigen presenting cell	leukocyte
prostate gland microvascular endothelial cell	blood vessel endothelial cell
prostate gland microvascular endothelial cell	endothelial cell of vascular tree
prostate gland microvascular endothelial cell	endothelial cell
prostate gland microvascular endothelial cell	epithelial cell
prostate gland microvascular endothelial cell	squamous epithelial cell
pulmonary artery endothelial cell	endothelial cell of artery
pulmonary artery endothelial cell	endothelial cell
pulmonary artery endothelial cell	epithelial cell
pulmonary artery endothelial cell	endothelial cell of vascular tree
pulmonary artery endothelial cell	blood vessel endothelial cell
pulmonary artery endothelial cell	squamous epithelial cell
pulmonary ionocyte	epithelial cell of lung
pulmonary ionocyte	epithelial cell
pulmonary ionocyte	ionocyte
radial glial cell	glial cell
radial glial cell	neural cell
regular atrial cardiac myocyte	cardiac muscle cell
regular atrial cardiac myocyte	muscle cell
regular atrial cardiac myocyte	contractile cell
regular ventricular cardiac myocyte	cardiac muscle cell

regular ventricular cardiac myocyte	muscle cell
regular ventricular cardiac myocyte	contractile cell
regulatory T cell	T cell
regulatory T cell	lymphocyte
regulatory T cell	leukocyte
respiratory basal cell	epithelial cell
respiratory basal cell	stem cell
respiratory basal cell	basal cell
respiratory goblet cell	goblet cell
respiratory goblet cell	glandular epithelial cell
respiratory goblet cell	epithelial cell
respiratory goblet cell	secretory cell
respiratory goblet cell	mucus secreting cell
retina horizontal cell	neuron
retina horizontal cell	neural cell
retinal bipolar neuron	neuron
retinal bipolar neuron	neural cell
retinal bipolar neuron	secretory cell
retinal blood vessel endothelial cell	retinal blood vessel endothelial cell
retinal blood vessel endothelial cell	endothelial cell of vascular tree
retinal blood vessel endothelial cell	endothelial cell
retinal blood vessel endothelial cell	epithelial cell
retinal blood vessel endothelial cell	squamous epithelial cell
retinal blood vessel endothelial cell	neural cell
retinal cone cell	neuron
retinal cone cell	neural cell
retinal ganglion cell	neuron
retinal ganglion cell	neural cell

retinal pigment epithelial cell	epithelial cell
retinal pigment epithelial cell	neural cell
retinal rod cell	neuron
retinal rod cell	neural cell
retinal rod cell	eye photoreceptor cell
Schwann cell	glial cell
Schwann cell	neural cell
serous cell of epithelium of bronchus	serous secreting cell
serous cell of epithelium of bronchus	secretory cell
serous cell of epithelium of bronchus	tracheobronchial serous cell
serous cell of epithelium of trachea	serous secreting cell
serous cell of epithelium of trachea	secretory cell
serous cell of epithelium of trachea	tracheobronchial serous cell
serous secreting cell	secretory cell
skeletal muscle fiber	muscle cell
skeletal muscle fiber	contractile cell
skeletal muscle fiber	cell of skeletal muscle
skeletal muscle satellite cell	cell of skeletal muscle
skeletal muscle satellite stem cell	stem cell
skeletal muscle satellite stem cell	skeletal muscle satellite cell
skeletal muscle satellite stem cell	cell of skeletal muscle
skin fibroblast	fibroblast
skin fibroblast	connective tissue cell
slow muscle cell	skeletal muscle fiber
slow muscle cell	muscle cell
slow muscle cell	contractile cell
slow muscle cell	cell of skeletal muscle
small intestine goblet cell	epithelial cell

small intestine goblet cell	glandular epithelial cell
small intestine goblet cell	goblet cell
small intestine goblet cell	secretory cell
small intestine goblet cell	mucus secreting cell
smooth muscle cell	muscle cell
smooth muscle cell	contractile cell
smooth muscle cell of prostate	smooth muscle cell
smooth muscle cell of prostate	muscle cell
smooth muscle cell of prostate	contractile cell
squamous epithelial cell	epithelial cell
stratified epithelial cell	epithelial cell
stromal cell	connective tissue cell
stromal cell of ovary	stromal cell
stromal cell of ovary	connective tissue cell
subcutaneous fat cell	fat cell
subcutaneous fat cell	connective tissue cell
T cell	lymphocyte
T cell	leukocyte
T follicular helper cell	CD4-positive helper T cell
T follicular helper cell	CD4-positive, alpha-beta T cell
T follicular helper cell	alpha-beta T cell
T follicular helper cell	T cell
T follicular helper cell	lymphocyte
T follicular helper cell	leukocyte
T follicular helper cell	stromal cell
T follicular helper cell	connective tissue cell
thymocyte	T cell
thymocyte	lymphocyte

thymocyte	leukocyte
tongue muscle cell	skeletal muscle fiber
tongue muscle cell	muscle cell
tongue muscle cell	contractile cell
tongue muscle cell	cell of skeletal muscle
tracheal goblet cell	epithelial cell
tracheal goblet cell	tracheobronchial goblet cell
tracheal goblet cell	glandular epithelial cell
tracheal goblet cell	goblet cell
tracheal goblet cell	respiratory goblet cell
tracheal goblet cell	secretory cell
tracheal goblet cell	mucus secreting cell
tracheobronchial goblet cell	epithelial cell
tracheobronchial goblet cell	glandular epithelial cell
tracheobronchial goblet cell	goblet cell
tracheobronchial goblet cell	respiratory goblet cell
tracheobronchial goblet cell	secretory cell
tracheobronchial goblet cell	mucus secreting cell
tracheobronchial serous cell	serous secreting cell
tracheobronchial serous cell	secretory cell
tracheobronchial smooth muscle cell	smooth muscle cell
tracheobronchial smooth muscle cell	muscle cell
tracheobronchial smooth muscle cell	contractile cell
transitional stage B cell	B cell
transitional stage B cell	lymphocyte
transitional stage B cell	leukocyte
type B pancreatic cell	epithelial cell
type B pancreatic cell	glandular epithelial cell

type B pancreatic cell	secretory cell
type I NK T cell	mature NK T cell
type I NK T cell	alpha-beta T cell
type I NK T cell	alveolar macrophage
type I NK T cell	amacrine cell
type I NK T cell	T cell
type I NK T cell	lymphocyte
type I NK T cell	leukocyte
type I pneumocyte	epithelial cell of alveolus of lung
type I pneumocyte	epithelial cell
type I pneumocyte	epithelial cell of lung
type II pneumocyte	epithelial cell of alveolus of lung
type II pneumocyte	epithelial cell
type II pneumocyte	epithelial cell of lung
uterine smooth muscle cell	smooth muscle cell
uterine smooth muscle cell	muscle cell
uterine smooth muscle cell	contractile cell
vascular associated smooth muscle cell	smooth muscle cell
vascular associated smooth muscle cell	muscle cell
vascular associated smooth muscle cell	contractile cell
vein endothelial cell	blood vessel endothelial cell
vein endothelial cell	endothelial cell of vascular tree
vein endothelial cell	endothelial cell
vein endothelial cell	epithelial cell
vein endothelial cell	squamous epithelial cell

Table S4. Evaluation of Hierarchical Correction Using AUC:

Cell Type	Fold 1	Fold 2	Fold 3	Fold 4	Fold 5	Fold 6	Fold 7	Fold 8	Fold 9	Fold 10
endothelial cell of hepatic sinusoid	na	na	na	na	na	na	na	na	na	na
endothelial cell	0.998	0.999	0.991	0.931	0.999	0.998	0.998	0.998	1.000	0.999
endothelial cell of lymphatic vessel	1.000	1.000	0.994	0.826	na	0.998	0.999	0.998	0.998	0.994
vein endothelial cell	na	0.992	na	0.939	na	0.995	na	0.988	0.996	0.979
endothelial cell of artery	na	0.996	na	0.985	na	0.997	na	0.994	0.995	0.987
capillary endothelial cell	na	0.996	na	0.973	na	0.996	na	0.995	0.993	0.991
retinal blood vessel endothelial cell	na	na	na	na	na	na	na	na	na	na
cardiac endothelial cell	0.267	na	na	0.218	na	na	0.101	0.224	na	na
endothelial cell of vascular tree	0.996	0.991	0.993	0.976	na	0.994	0.995	0.992	0.997	0.996
gut endothelial cell	na	na	na	na	na	na	na	na	na	na
blood vessel endothelial cell	0.981	0.992	0.997	0.973	na	0.995	0.977	0.977	0.997	0.994
lung microvascular endothelial cell	na	na	na	na	na	na	na	na	na	na
slow muscle cell	0.763	na	0.999	na	na	na	0.843	0.580	na	na
skeletal muscle fiber	0.860	na	0.992	na	na	na	0.932	0.969	na	na
skin fibroblast	na	na	na	na	na	na	na	na	na	na
fast muscle cell	0.859	na	0.992	na	na	na	0.968	0.986	na	na
contractile cell	0.986	0.991	0.848	0.990	0.995	0.990	0.979	0.980	0.997	0.987
professional antigen presenting cell	0.979	0.983	0.975	0.993	0.953	0.986	0.991	0.895	0.589	0.957
dendritic cell	0.979	0.984	1.000	0.991	0.986	0.987	0.965	0.975	0.726	0.982
mast cell	0.988	0.998	0.999	0.999	0.987	0.999	0.981	0.985	1.000	0.994
subcutaneous fat cell	na	na	na	na	na	na	na	na	na	na
macrophage	0.978	0.995	na	0.997	0.969	0.995	0.990	0.956	0.999	0.997
Schwann cell	0.448	na	0.926	na	na	na	0.450	0.253	na	na
skeletal muscle satellite cell	0.955	na	0.950	na	na	na	0.879	0.961	na	na
neutrophil	0.747	na	na	na	na	na	0.903	na	na	na
T cell	0.999	0.928	0.965	0.992	0.903	0.992	0.983	0.857	0.627	0.994

mature NK T cell	0.981	0.928	na	0.878	na	0.958	0.983	0.531	na	na
luminal epithelial cell of mammary gland	0.902	na	0.774	na	na	0.843	na	0.773	na	na
myoepithelial cell of mammary gland	na	na	na	na	na	na	na	na	na	na
squamous epithelial cell	0.900	0.991	0.627	0.973	na	0.966	0.619	0.969	0.997	0.970
basal cell	0.839	na	0.788	na	na	0.906	0.861	0.883	0.994	0.938
basal epithelial cell of prostatic duct	na	na	na	na	na	na	na	na	na	na
glandular epithelial cell	0.947	na	0.497	na	na	0.716	0.656	0.906	0.971	0.966
fibroblast	0.996	0.984	0.900	0.976	0.998	0.947	0.994	0.995	0.986	0.999
fibroblast of cardiac tissue	0.394	na	0.177	na	na	na	0.288	0.496	na	na
B cell	0.999	0.998	1.000	0.998	0.997	0.999	0.999	0.993	0.982	0.998
neuroendocrine cell	0.964	na	na	na	na	na	0.932	0.891	0.997	0.970
myeloid dendritic cell	0.917	na	na	na	na	na	0.957	0.971	0.989	0.953
smooth muscle cell of prostate	0.934	na	na	na	na	na	0.971	na	na	na
enteric smooth muscle cell	na	na	na	na	na	na	na	na	na	na
neuron	1.000	na	0.999	na	na	0.930	0.770	0.990	na	na
interstitial cell of Cajal	na	na	na	na	na	na	na	na	na	na
cardiac muscle cell	0.991	0.992	0.893	0.991	na	0.994	0.994	0.961	1.000	na
smooth muscle cell	0.977	0.986	0.918	0.991	0.993	0.991	0.975	0.989	0.997	0.990
epithelial cell of alveolus of lung	0.961	na	0.993	na	na	0.991	0.989	0.990	0.999	0.999
respiratory basal cell	0.655	na	na	na	na	na	0.449	0.633	0.977	0.693
alveolar macrophage	0.972	na	na	na	na	na	0.892	0.526	0.997	0.979
epithelial cell of prostate	0.883	na	0.743	na	na	0.787	0.903	0.925	na	na
elicited macrophage	0.929	na	na	na	na	0.267	0.919	0.951	0.959	0.265
luminal cell of prostate epithelium	0.983	na	0.999	na	na	0.997	0.976	0.950	na	na
epithelial cell of sweat gland	na	na	na	na	na	na	na	na	na	na
basal cell of epidermis	na	na	na	na	na	na	na	na	na	na

epidermal cell	0.853	na	0.171	na	na	0.226	na	0.917	na	na
sebaceous gland cell	na	na	na	na	na	na	na	na	na	na
keratinocyte	0.836	na	0.188	na	na	0.420	na	0.810	na	na
melanocyte of skin	na	na	na	na	na	na	na	na	na	na
epidermal Langerhans cell	na	na	na	na	na	na	na	na	na	na
monocyte	0.992	0.993	na	0.995	0.995	0.993	na	0.964	0.814	0.989
liver dendritic cell	na	na	na	na	na	na	na	na	na	na
plasma cell	0.998	0.998	na	0.997	0.997	0.999	na	0.875	0.992	0.989
CD8-positive, alpha-beta T cell	0.995	0.959	0.994	0.938	0.925	0.979	0.996	0.912	0.485	0.991
CD4-positive, alpha-beta T cell	0.997	0.974	0.960	0.974	0.939	0.993	0.948	0.891	0.682	0.987
double-positive, alpha-beta thymocyte	na	0.319	na	0.549	0.449	na	na	na	na	na
erythrocyte	na	0.988	na	0.974	0.920	na	na	0.831	0.246	na
CD4-positive, alpha-beta memory T cell	0.998	0.783	na	0.849	0.611	na	na	0.724	0.266	na
CD8-positive, alpha-beta cytokine secreting effector T cell	na	na	na	na	na	na	na	na	na	na
classical monocyte	na	na	na	na	na	na	na	0.973	0.990	0.960
naive B cell	na	0.875	na	0.893	0.770	na	na	0.927	0.257	na
memory B cell	na	0.990	na	0.996	0.956	na	na	0.952	0.263	na
type I NK T cell	na	na	na	na	na	na	na	na	na	na
CD141-positive myeloid dendritic cell	na	na	na	na	na	na	na	na	na	na
platelet	0.996	0.994	na	na	0.990	0.995	na	0.959	0.366	na
Naive thymus-derived CD4-positive, alpha-beta T cell	0.997	0.983	na	na	0.950	0.992	na	0.821	0.403	na
non-classical monocyte	na	na	na	na	na	na	na	0.942	0.522	0.964
hematopoietic stem cell	0.991	0.971	na	na	0.823	0.983	na	0.945	na	na
plasmacytoid dendritic cell	0.999	0.998	na	1.000	0.996	1.000	na	0.971	0.440	0.999
basophil	na	na	na	na	na	na	na	na	na	na

effector CD8-positive, alpha-beta T cell	na	na	na	na	na	na	na	na	na	na
effector CD4-positive, alpha-beta T cell	na	na	na	na	na	na	na	na	na	na
innate lymphoid cell	0.999	0.978	na	0.956	0.928	0.996	na	0.580	0.647	0.999
regulatory T cell	0.998	0.875	na	0.952	0.781	0.988	na	0.924	0.489	na
CD1c-positive myeloid dendritic cell	na	na	na	na	na	na	na	0.981	0.993	0.961
intermediate monocyte	na	na	na	na	na	na	na	na	na	na
mature conventional dendritic cell	na	na	na	na	na	na	na	na	na	na
CD4-positive helper T cell	0.996	0.975	na	na	0.960	0.988	na	0.657	0.999	na
CD8-positive, alpha-beta memory T cell	0.999	0.901	na	0.938	0.841	na	na	0.859	0.417	na
naive thymus-derived CD8-positive, alpha-beta T cell	0.999	na	na	na	na	na	na	0.707	0.342	na
leukocyte	0.999	0.998	0.947	0.998	0.996	0.992	0.990	0.977	0.997	0.995
microglial cell	na	na	na	na	na	na	na	na	na	na
erythroid lineage cell	na	0.998	na	0.999	0.988	na	na	0.994	0.276	na
mesenchymal stem cell	na	na	0.910	na	na	na	na	0.825	na	na
CD8-positive, alpha-beta cytotoxic T cell	0.958	0.952	0.994	0.990	na	0.856	0.989	0.334	na	0.955
Langerhans cell	0.852	na	na	na	na	na	0.712	na	na	na
erythroid progenitor cell	na	na	na	na	na	na	na	na	na	na
granulocyte	0.718	na	na	na	na	na	0.873	na	na	na
common myeloid progenitor	na	na	na	na	na	na	na	na	na	na
myeloid cell	0.963	0.994	0.972	0.996	0.979	0.995	0.926	0.961	0.982	0.999
plasmablast	0.994	0.996	na	na	0.992	0.996	na	0.913	0.348	na
naive regulatory T cell	na	na	na	na	na	na	na	na	na	na
T follicular helper cell	0.994	0.977	na	na	0.905	0.989	na	0.680	1.000	na
DN4 thymocyte	na	na	na	na	na	na	na	na	na	na
DN3 thymocyte	na	na	na	na	na	na	na	na	na	na

DN1 thymic pro-T cell	na	na	na	na	na	na	na	na	na	na
immature natural killer cell	na	na	na	na	na	na	na	na	na	na
thymocyte	na	0.832	na	0.965	0.837	na	na	0.751	0.264	na
natural killer cell	0.999	0.987	na	0.982	0.987	0.998	na	0.922	0.692	0.999
activated CD8-positive, alpha-beta T cell	0.979	na	na	0.986	0.965	na	na	na	na	na
follicular B cell	na	na	na	na	na	na	na	na	na	na
CD4-positive, CD25-positive, alpha-beta regulatory T cell	0.967	0.978	na	na	0.876	0.856	na	0.791	na	na
lymphocyte	1.000	0.997	0.974	0.998	0.993	0.997	0.991	0.978	0.995	0.998
CD8-alpha alpha positive, gamma-delta intraepithelial T cell	na	na	na	na	na	na	na	na	na	na
conventional dendritic cell	0.955	0.997	na	na	0.992	0.998	0.968	0.982	0.760	0.982
IgM plasma cell	na	na	na	na	na	na	na	na	na	na
IgG plasma cell	na	na	na	na	na	na	na	na	na	na
native cell	0.799	0.516	na	0.529	0.947	0.514	na	na	na	na
basal cell of prostate epithelium	0.827	na	0.699	na	na	0.811	0.896	0.847	na	na
secretory cell	0.973	0.999	0.728	0.999	0.989	0.809	0.628	0.969	0.961	0.981
fibroblast of connective tissue of glandular part of prostate	na	na	na	na	na	na	na	na	na	na
epithelial cell of urethra	na	na	na	na	na	na	na	na	na	na
fibroblast of connective tissue of nonglandular part of prostate	na	na	na	na	na	na	na	na	na	na
prostate gland microvascular endothelial cell	na	na	na	na	na	na	na	na	na	na
pericyte cell	na	0.998	0.745	0.996	na	0.998	na	0.998	1.000	0.998
vascular associated smooth muscle cell	na	0.891	0.884	0.917	0.946	na	na	0.240	na	na
CD4-positive, alpha-beta thymocyte	na	na	na	na	na	na	na	na	na	na
CD8-positive, alpha-beta thymocyte	na	na	na	na	na	na	na	na	na	na

stromal cell	0.986	0.950	0.838	na	0.929	0.919	0.780	0.524	0.940	0.618
adventitial cell	na	na	na	na	na	na	na	na	na	na
tongue muscle cell	na	na	na	na	na	na	na	na	na	na
fibroblast of breast	na	na	na	na	na	na	na	na	na	na
myometrial cell	na	na	na	na	na	na	na	na	na	na
Muller cell	na	na	na	na	na	na	na	na	na	na
melanocyte	0.908	na	0.953	na	na	na	0.816	0.841	na	na
retinal bipolar neuron	na	na	0.025	na	na	0.783	0.730	na	na	na
radial glial cell	na	na	na	na	na	na	na	na	na	na
retina horizontal cell	na	na	0.381	na	na	0.497	0.768	na	na	na
myofibroblast cell	na	na	0.676	na	na	na	na	na	0.934	0.939
muscle cell	0.987	0.990	0.828	0.989	0.991	0.989	0.985	0.986	0.997	0.990
pancreatic stellate cell	na	na	na	na	na	na	na	na	na	na
skeletal muscle satellite stem cell	na	na	na	na	na	na	na	na	na	na
tendon cell	na	na	na	na	na	na	na	na	na	na
mesothelial cell	na	0.999	0.939	1.000	na	0.997	0.938	1.000	0.998	0.983
fat cell	1.000	0.987	0.670	0.983	na	0.971	1.000	1.000	na	na
retinal ganglion cell	na	na	na	na	na	na	na	na	na	na
cell of skeletal muscle	0.865	na	0.977	na	na	na	0.854	0.965	na	na
bronchial smooth muscle cell	na	na	na	na	na	na	na	na	na	na
connective tissue cell	0.991	0.981	0.983	0.978	0.985	0.977	0.992	0.990	0.999	0.985
hepatocyte	na	na	na	na	na	na	na	na	na	na
intrahepatic cholangiocyte	na	na	na	na	na	na	na	na	na	na
tracheal goblet cell	na	na	na	na	na	na	na	na	na	na
ciliated cell	na	na	0.820	na	na	0.808	0.761	na	0.997	0.999
ionocyte	na	na	0.990	na	na	0.996	0.994	na	0.990	0.982
mucus secreting cell	0.986	na	0.803	na	na	0.818	0.887	na	0.981	0.970
serous cell of epithelium of trachea	na	na	na	na	na	na	na	na	na	na

acinar cell of salivary gland	na	na	na	na	na	na	na	na	na	na
duct epithelial cell	0.867	na	0.578	na	na	0.669	na	0.896	na	na
myoepithelial cell	0.867	na	0.780	na	na	0.747	na	0.815	na	na
epithelial cell	0.987	0.998	0.800	0.946	0.991	0.852	0.946	0.971	0.997	0.986
epithelial cell of uterus	na	na	na	na	na	na	na	na	na	na
ciliated epithelial cell	na	na	0.810	na	na	0.649	0.571	na	0.993	0.983
conjunctival epithelial cell	na	na	na	na	na	na	na	na	na	na
eye photoreceptor cell	na	na	0.999	na	na	na	na	na	na	na
stem cell	0.872	0.974	0.621	na	0.911	0.872	0.850	0.836	0.994	0.921
epithelial cell of lacrimal sac	na	na	na	na	na	na	na	na	na	na
keratocyte	na	na	na	na	na	na	na	na	na	na
retinal pigment epithelial cell	na	na	na	na	na	na	na	na	na	na
corneal epithelial cell	na	na	na	na	na	na	na	na	na	na
surface ectodermal cell	na	na	na	na	na	na	na	na	na	na
pigmented ciliary epithelial cell	na	na	na	na	na	na	na	na	na	na
pancreatic acinar cell	na	na	na	na	na	na	na	na	na	na
pancreatic ductal cell	na	na	na	na	na	na	na	na	na	na
type B pancreatic cell	na	na	na	na	na	na	na	na	na	na
club cell	na	na	0.284	na	na	0.257	0.710	na	0.941	0.874
salivary gland cell	na	na	na	na	na	na	na	na	na	na
medullary thymic epithelial cell	na	0.413	0.369	0.507	0.215	0.111	0.200	0.659	na	na
bladder urothelial cell	na	na	na	na	na	na	na	na	na	na
enterocyte of epithelium of large intestine	na	na	na	na	na	na	na	na	na	na
large intestine goblet cell	na	na	na	na	na	na	na	na	na	na
paneth cell of colon	na	na	na	na	na	na	na	na	na	na
transit amplifying cell of colon	na	na	na	na	na	na	na	na	na	na
intestinal crypt stem cell of large intestine	na	na	na	na	na	na	na	na	na	na

intestinal enteroendocrine cell	na	na	na	na	na	na	na	na	na	na
intestinal tuft cell	na	na	na	na	na	na	na	na	na	na
type II pneumocyte	na	na	0.968	na	na	0.994	0.991	na	0.998	0.997
respiratory goblet cell	na	na	0.798	na	na	0.790	0.883	na	0.979	0.961
enterocyte of epithelium of small intestine	na	na	na	na	na	na	na	na	na	na
transit amplifying cell of small intestine	na	na	na	na	na	na	na	na	na	na
small intestine goblet cell	na	na	na	na	na	na	na	na	na	na
paneth cell of epithelium of small intestine	na	na	na	na	na	na	na	na	na	na
intestinal crypt stem cell of small intestine	na	na	na	na	na	na	na	na	na	na
kidney epithelial cell	na	na	na	na	na	na	na	na	na	na
enterocyte	na	na	na	na	na	na	na	na	na	na
intestinal crypt stem cell	na	na	na	na	na	na	na	na	na	na
goblet cell	na	na	0.808	na	na	0.789	0.880	na	0.977	0.970
lung ciliated cell	na	na	na	na	na	na	na	na	na	na
serous cell of epithelium of bronchus	na	na	na	na	na	na	na	na	na	na
type I pneumocyte	na	na	0.886	na	na	0.855	0.883	na	0.988	0.495
pulmonary ionocyte	na	na	na	na	na	na	na	na	na	na
pancreatic PP cell	na	na	na	na	na	na	na	na	na	na
pancreatic A cell	na	na	na	na	na	na	na	na	na	na
pancreatic D cell	na	na	na	na	na	na	na	na	na	na
duodenum glandular cell	na	na	na	na	na	na	na	na	na	na
central memory CD4-positive, alpha-beta T cell	na	na	na	na	na	na	na	na	na	na
effector memory CD8-positive, alpha-beta T cell	na	na	na	na	na	na	na	na	na	na
central memory CD8-positive, alpha-beta T cell	na	na	na	na	na	na	na	na	na	na

effector memory CD4-positive, alpha-beta T cell	0.999	na	na	na	na	na	na	na	0.545	na
CD16-negative, CD56-bright natural killer cell, human	1.000	na	na	na	na	na	na	na	0.675	na
transitional stage B cell	na	na	na	na	na	na	na	na	na	na
CD14-low, CD16-positive monocyte	na	na	na	na	na	na	na	na	na	na
mucosal invariant T cell	0.996	na	na	na	na	na	na	na	0.524	na
CD14-positive monocyte	na	0.985	na	0.994	na	0.983	na	na	0.340	na
gamma-delta T cell	0.993	0.844	na	0.794	0.696	0.996	na	0.826	0.538	na
CD4-positive, alpha-beta cytotoxic T cell	na	0.916	na	0.962	na	0.885	na	na	0.281	na
double negative thymocyte	na	0.704	na	0.909	0.680	na	na	0.760	0.255	na
hematopoietic precursor cell	0.994	0.983	na	na	0.926	0.994	na	0.483	0.255	na
peripheral blood mononuclear cell	na	na	na	na	na	na	na	na	na	na
CD8-alpha-alpha-positive, alpha-beta intraepithelial T cell	na	na	na	na	na	na	na	na	na	na
alpha-beta T cell	0.995	0.861	0.977	0.942	0.751	0.991	0.977	0.774	0.733	0.992
precursor B cell	na	0.173	na	0.280	0.901	na	na	0.217	na	na
progenitor cell	0.934	0.984	na	0.973	0.957	0.990	0.902	0.897	0.822	0.990
early T lineage precursor	na	na	na	na	na	na	na	na	na	na
group 3 innate lymphoid cell	1.000	0.854	na	0.883	0.730	na	na	0.887	1.000	na
epithelial cell of thymus	na	0.464	0.696	0.720	0.516	0.653	0.358	0.728	na	na
cortical thymic epithelial cell	na	na	na	na	na	na	na	na	na	na
megakaryocyte	na	0.953	na	0.992	0.995	na	na	0.996	na	na
CD8-positive, alpha-beta memory T cell, CD45RO-positive	0.999	na	na	na	na	na	na	na	0.288	na
effector memory CD8-positive, alpha-beta T cell, terminally differentiated	0.998	na	na	na	na	na	na	na	0.999	na
animal cell	na	na	na	na	na	na	na	na	na	na

CD16-positive, CD56-dim natural killer cell, human	1.000	na	na	na	na	na	na	na	1.000	na
dendritic cell, human	na	na	na	na	na	na	na	na	na	na
germinal center B cell	na	na	na	na	na	na	na	na	na	na
pro-B cell	na	na	na	na	na	na	na	na	na	na
stromal cell of ovary	na	na	na	na	na	na	na	na	na	na
supportive cell	na	na	0.336	na	na	0.359	na	na	0.869	na
eukaryotic cell	na	na	na	na	na	na	na	na	na	na
uterine smooth muscle cell	na	na	0.906	na	na	0.983	na	na	0.973	na
regular ventricular cardiac myocyte	0.998	0.990	na	0.989	na	0.994	0.998	na	1.000	na
stratified epithelial cell	0.939	na	0.677	na	na	0.602	na	0.616	na	0.908
epithelial cell of stratum germinativum of esophagus	na	na	na	na	na	na	na	na	na	na
phagocyte	0.981	0.991	0.983	0.995	0.969	0.992	0.987	0.945	0.997	0.993
immature B cell	na	na	na	na	na	na	na	na	na	na
retinal rod cell	na	na	na	na	na	na	na	na	na	na
retinal cone cell	na	na	na	na	na	na	na	na	na	na
glial cell	0.920	na	0.538	na	na	0.914	0.732	0.900	na	na
amacrine cell	na	na	na	na	na	0.497	0.543	0.199	na	na
pulmonary artery endothelial cell	na	na	na	na	na	na	na	na	na	na
bronchus fibroblast of lung	na	na	na	na	na	na	na	na	na	na
ciliated columnar cell of tracheobronchial tree	na	na	na	na	na	0.575	0.707	na	0.985	0.903
nasal mucosa goblet cell	na	na	na	na	na	0.585	0.607	na	0.933	0.889
tracheobronchial serous cell	na	na	0.932	na	na	0.809	0.994	na	0.973	0.907
fibroblast of lung	na	na	na	na	na	na	na	na	na	na
tracheobronchial smooth muscle cell	na	na	0.976	na	na	na	na	na	0.839	0.624
multi-ciliated epithelial cell	na	na	0.553	na	na	0.836	0.895	na	0.995	0.930
bronchial goblet cell	na	na	na	na	na	na	na	na	na	na

lung neuroendocrine cell	na	na	na	na	na	na	na	na	na	na
epithelial cell of lung	0.957	na	0.988	0.560	na	0.988	0.993	0.985	0.999	0.998
lung macrophage	0.945	na	na	na	na	na	0.963	0.879	0.995	0.980
tracheobronchial goblet cell	na	na	0.948	na	na	0.885	0.918	na	0.962	0.745
acinar cell	na	na	0.633	na	na	0.578	0.572	na	0.981	0.853
serous secreting cell	na	na	0.848	na	na	0.760	0.973	na	0.978	0.863
brush cell of trachebronchial tree	na	na	na	na	na	na	na	na	na	na
epicardial adipocyte	na	na	na	na	na	na	na	na	na	na
neural cell	0.999	0.977	0.909	0.934	na	0.943	0.995	0.928	na	na
CD14-positive, CD16-positive monocyte	na	na	na	na	na	na	na	na	na	na
activated CD4-positive, alpha-beta T cell	na	na	na	na	na	na	na	na	na	na
regular atrial cardiac myocyte	na	na	na	na	na	na	na	na	na	na

Table S5. Evaluation of the model using AUC on the lung atlas data published in 2023

Cell type	AUC
elicited macrophage	0.2685675322
stromal cell	0.3353826191
type I pneumocyte	0.5052777464
tracheobronchial smooth muscle cell	0.6250444438
respiratory basal cell	0.7240044178
tracheobronchial goblet cell	0.7434875376
acinar cell	0.8432369629
serous secreting cell	0.8453848703
club cell	0.8614955385

nasal mucosa goblet cell	0.8922806837
tracheobronchial serous cell	0.8992880418
ciliated columnar cell of tracheobronchial tree	0.9027628335
multi-ciliated epithelial cell	0.9288761238
myofibroblast cell	0.9323367753
myeloid dendritic cell	0.9515293428
professional antigen presenting cell	0.95269827
respiratory goblet cell	0.9554767557
classical monocyte	0.9561722708
glandular epithelial cell	0.9606803859
CD1c-positive myeloid dendritic cell	0.9617625564
non-classical monocyte	0.9626002949
stem cell	0.9648953189
goblet cell	0.9655851961
mucus secreting cell	0.9658673339
basal cell	0.9668242113
alveolar macrophage	0.9757958025
neuroendocrine cell	0.9780481875
secretory cell	0.9784147482
lung macrophage	0.9786234769
ionocyte	0.9797439518
dendritic cell	0.9798843417
conventional dendritic cell	0.9824441459

ciliated epithelial cell	0.9825337232
mesothelial cell	0.9844914369
contractile cell	0.985982021
epithelial cell	0.986972439
connective tissue cell	0.9872446519
endothelial cell of artery	0.9872503605
plasma cell	0.9879646519
muscle cell	0.9889768123
monocyte	0.9893160745
CD4-positive, alpha-beta T cell	0.9894947518
smooth muscle cell	0.9896376113
CD8-positive, alpha-beta T cell	0.9904884055
progenitor cell	0.9913424012
squamous epithelial cell	0.9915159509
vein endothelial cell	0.9919554728
phagocyte	0.9923144792
capillary endothelial cell	0.9930028405
mast cell	0.993282361
alpha-beta T cell	0.9935462544
leukocyte	0.9943017207
endothelial cell of lymphatic vessel	0.9947019921
T cell	0.9950725781
blood vessel endothelial cell	0.9962465029
type II pneumocyte	0.9964505465
macrophage	0.9967123231
endothelial cell of vascular tree	0.9969493915

B cell	0.9977572332
epithelial cell of lung	0.9981579068
lymphocyte	0.9983684286
pericyte cell	0.9985407941
ciliated cell	0.9985579678
epithelial cell of alveolus of lung	0.9986903477
plasmacytoid dendritic cell	0.9989711573
innate lymphoid cell	0.9990903605
myeloid cell	0.9991071041
natural killer cell	0.9992415206
fibroblast	0.9993082627
endothelial cell	0.9995163468
